# The role of corruption in global food systems: a systematic scoping review

**DOI:** 10.1186/s12992-024-01054-8

**Published:** 2024-06-15

**Authors:** Anastassia Demeshko, Chloe Clifford Astbury, Kirsten M. Lee, Janielle Clarke, Katherine Cullerton, Tarra L. Penney

**Affiliations:** 1 Global Food Systems & Policy Research, School of Global Health, York University, Toronto, ON Canada; 2 Dahdaleh Institute for Global Health Research, York University, Toronto, ON Canada; 3 School of Public Health, University of Queensland, Brisbane, QLD Australia

**Keywords:** Corruption, Global food system

## Abstract

**Background:**

Corruption exists at all levels of our global society and is a potential threat to food security, food safety, equity, and social justice. However, there is a knowledge gap in the role and impact of corruption within the context of the global food system. We aimed to systematically review empirical literature focused on corruption in the global food system to examine how it is characterized, the actors involved, its potential impacts, and the solutions that have been proposed to address corruption in the food system.

**Methods:**

We used a systematic scoping review methodology. Terms combining corruption and the food system were searched in Scopus, PubMed, Web of Science, PsycInfo and Econlit, in October 2021. Two screeners applied *a priori* selection criteria to screen the articles at the title and abstract and full-text levels. Data was extracted into a charting form and thematically synthesized to describe the types of corruption in the food system, the actors involved, how corruption impacts the food system, and potential solutions. Sankey diagrams and narrative summaries were developed to summarize the included studies and findings.

**Results:**

From the 238 included records, five main types of corruption were identified in the global food system: bureaucratic corruption, fraud, bribery, organized crime, and corporate political activity. These different types of corruption spanned across various food system areas, from policy and governance structures to food environments, and involved a wide range of actors. More powerful actors like those in public and private sectors tended to instigate corruption in the food system, while community members and primary producers tended to be impacted by it. The impacts of corruption were mostly negative and corruption was found to undermine food system governance and regulatory structures; threaten health, safety, and food security; and lead or contribute to environmental degradation, economic loss, erosion of trust, social inequities, and decreased agricultural productivity. While solution-oriented literature was limited, the essential role of strong governance,  use of technology and predictive modelling methods to improve detection of corruption, and organizational approaches to problem solving were identified.

**Conclusion:**

Our review findings provide researchers and policymakers with a comprehensive overview of corruption in the global food system, providing insights to inform a more holistic approach to addressing the issue. Addressing corruption in the food system is an essential element of supporting the transition to a more healthy, equitable and sustainable global food system.

**Supplementary Information:**

The online version contains supplementary material available at 10.1186/s12992-024-01054-8.

## Introduction

Corruption is a complex phenomenon which takes many forms and exists at all levels of global society [1]. Within the global food system, there is limited understanding of the types of corruption that exist, the actors involved, and whether the potential impacts might disrupt efforts to transition to healthy, sustainable, and equitable food systems [2].

## The need for a systems approach for healthy, sustainable, and equitable food systems

The Food and Agriculture Organization defines the food system as encompassing “*the entire range of actors and their interlinked value-adding activities involved in the production, aggregation, processing, distribution, consumption and disposal of food products that originate from agriculture, forestry or fisheries, and parts of the broader economic, societal and natural environments in which they are embedded*” [3]. From production to consumption, the productivity and sustainability of the global food system are interconnected with policy and governance structures and systems that support food production (e.g., ecological, economic or health systems that food supply chains depend on) [4]. In turn, these directly and indirectly affect the food supply chains, food environments, consumer behaviors, diets, and health outcomes contained within the food system [3, 5].

The current food system is failing to provide nutritious foods for all [6]. Inextricably linked to issues of health, humanitarianism, and environmental sustainability [7, 8], the food system is associated with complex challenges such as poverty, non-communicable disease, environmental degradation, and economic downturns [9]. More than 800 million people experience hunger [9], over two billion experience micronutrient deficiencies [10], and almost two billion live with overweight or obesity [11]. While enough food is produced to feed the world, 931 million tons of food were wasted in 2019–17% of all food produced [12]. Food systems are essential to meet the Sustainable Development Goals (SDGs), including ‘zero hunger’ and ‘responsible consumption and production’ [3].

Given the complexity of food system challenges, there has been a call for systems approaches to guide a global transition to healthy, sustainable and equitable food systems [3]. A systems approach recognizes the totality of food system components and drivers, which may help to address the limitations of previous efforts to improve food security and nutrition, such as taking a production-focused approach that aims to increase food supply [3, 4, 13]. While this approach might allow systemic challenges, such as corruption, to be holistically conceptualized, these challenges can vary in their presentation, drivers, and impacts across the food system [6, 7].

## The challenge of corruption in the global food system

Corruption can be defined as the abuse of entrusted power, usually for the purpose of political, financial, or personal gain [1]. In its most common forms, corruption can occur as bribery, theft, nepotism, exploitation of conflicting interests, organized crime, legislative capture, extortion, improper political contributions, and poor governance [14]. Corruption has been shown to be a primary barrier for nations in meeting SDGs [15, 16]. However, although we know that corruption is present throughout society, little attention has been allocated to understanding its role in the context of improving global food systems in efforts to support health, the environment, and equity.

Given that corruption varies in type, activity, and between sectors, it is critical to develop context-specific understanding of how it operates in the food system [17–19]. Explicit acts of food system corruption have been identified, including public officials accepting bribes and participating in organized crime [20, 21]. Experts have also introduced the idea of ‘legal corruption’ [17, 22], which includes widespread practices in food policy and research such as unreported conflict of interest with the food and beverage industry [17]. Corruption is also interspersed in the functioning of society and therefore, difficult to eradicate given the role it plays in daily life [23–25]. For example, in some countries, corruption has become essential for ensuring jobs and farm loans can be secured. Understanding corruption in the global food system can inform anti-corruption policies and programs that minimize further impacts on vulnerable actors. Therefore, there is a need to understand corruption in the context of the global food system and address the knowledge gap in how we can integrate anti-corruption measures to support a food system transition [17, 26].

## Aims

We aimed to systematically review literature focused on corruption in the global food system to understand how it is characterized, the actors involved, whether and how corruption impacts the food system, and potential solutions to corruption in the food system.

### Methods

A systematic scoping review of peer-reviewed literature was conducted to investigate corruption in the global food system. The five-stage scoping review framework devised by Arksey and O’Malley, and refined by Levac et al., was used to identify and summarize the literature on this topic [27, 28]. The methodology and reporting were directed by the ‘Preferred Reporting Items for Systematic Reviews and Meta-Analyses for Scoping Reviews’ guidelines [29].

### Stage 1: identifying the research question

Informed by our study aims, our research questions were:

1. How is corruption in the food system characterized in the peer-reviewed literature?
2. What actors are involved in corruption in the food system?
3. How does corruption impact the food system?
4. What solutions have been proposed to address corruption in the food system?

### Stage 2: identifying relevant studies

Five electronic databases (Scopus, PubMed, Web of Science, PsycInfo, and Econlit) were systematically searched in October 2021 to identify the relevant literature for the scoping review. The main concepts of the research question informed the search strategy. These concepts were guided by the ‘Population, Concept, Context’ Framework established by the Joanna Briggs Institute (Table 1) [30]. Titles, abstracts, and keywords within the electronic databases were searched (see Supplementary File 1 for the full search strategy).

**Table 1:** Conceptual breakdown of the research question guided by the ‘population, concept, and context’ framework

**Population**	All actors within the food system
**Concept**	Influence of and impact of corruption in all its forms
**Context**	Global food system and governance

### Stage 3: study selection

Records identified through the database searches were collated and screened using *Covidence* reference management software [31]. All duplicates were removed. To select the relevant papers, the eligibility criteria presented in Table 2 were used.

**Table 2:** Eligibility criteria used for record screening

Inclusion	Exclusion
• Full-text peer-reviewed articles from any country or region, that are available in English. All methods and study designs will be considered; and • Corruption-related literature referring to or focusing on food systems (including papers that have subsections looking at corruption in the specific food system context).	• Clinical populations (i.e., diabetes, cardiovascular disease, etc.), non-human studies, physical science context (i.e., cellular and genetic studies, assays, laboratory setting, analytical food testing or detection techniques). • Corruptive strategies or psychological theory (i.e., bribery as a parental feeding strategy or foundations of corruptive behavior like greed); papers that were not focused on the food system and corruption; corruption of tools or technical processes. • Grey literature, editorials, book chapters, conference proceedings, commentaries, letters, reviews theoretical modelling studies. • Based on data collected before 1986. The start date was chosen as this was the year the Ottawa Charter was released, recommending a focus on healthy public policy as an effective strategy for health promotion.

A modified double screening process was used. First, AD and CCA independently screened an initial set of 100 titles and abstracts. Results were compared to ensure consistency in decisions around study eligibility, and disagreements were resolved through consensus. This process was repeated until an acceptable level of agreement (> 90%) was reached. The remaining records were screened by AD. AD and CCA screened 50% of title and abstracts before moving to single screening. Following this, full-text double screening was undertaken by AD and CCA on all articles, and conflicts were resolved by consensus. As recommended by published guidelines, the list of included studies was refined iteratively throughout the selection process [27, 28].

#### Stage 4: charting the data

Two researchers (AD and JC) extracted data using a data charting form (see Supplementary File 2), focusing on key study characteristics including the country context and area of the food system in which corruption occurred; type of corruption explored in the study; stakeholders involved; impacts of corruption; and any potential solutions proposed. In line with the Arksey and O’Malley scoping review guidelines, we trialed the data charting form with ten records, making revisions as needed to ensure the data was appropriately addressing the research questions. Amendments to the charting form involved broadening and simplifying the prompts for data extraction. This was due to the heterogeneity of study types, which made sections of the initial form inapplicable to some studies. In line with scoping review guidelines, a formal quality assessment of the records was not conducted.

#### Stage 5: collating, summarizing, and reporting the results

To summarize this large and heterogenous data set, we used both qualitative and quantitative approaches. Based on a review of the charted data, we developed and defined categories (Table 3) to summarize the included studies, drawing on relevant frameworks and definitions from the literature focused on types of corruption (e.g., Transparency International’s database, the concept of legal corruption, Corporate Political Activity framework) [32–35]; food system actors [36, 37]; and areas of the food system [5, 10]. We used the categories described in Table 3, as well as narrative summaries developed through qualitative content analysis [38] and visual summaries in the form of Sankey diagrams, to answer our research questions:

### How is corruption in the food system characterized in the peer-reviewed literature?

We characterized studies as focusing on one or more of the five corruption types (Table 3). For each type, we reviewed relevant summaries, narratively summarizing examples of this corruption type occurring within the food system, as well as measurement and data collection approaches.

### What actors are involved in corruption in the food system?

In describing the food system actors involved with corruption in each study, we identified two roles: instigators of corruption and those impacted by corruption. We categorized each of our included studies by the food system area in which corruption occurred, and the actors who instigated or were impacted by corruption. We summarized this information using a Sankey diagram to illustrate the concentration of corruption in particular food system areas, as well as the flow of corruption from instigators to those impacted. The Sankey diagram was developed using an open-source online tool, *SankeyMATIC* [39]. Sankey diagrams have been suggested as a useful tool to present patterns of evidence in systematic reviews, particularly when data is complex and heterogenous [40]; as was the case for our dataset. A Sankey diagram consists of nodes and their connecting flows (e.g., flows of information, resources, or characteristics) within a process or network [40, 41]. In our Sankey diagrams, the nodes represent the areas of interest in the review synthesis process, while the flows represent the number of studies in which a concept was identified. The width of each flow is proportional to the total number of times each concept was identified within the literature, and the intersection between different study characteristics (e.g., how many studies reported on corruption perpetuated by government officials and, of these, how many reported impacts on farmers versus consumers versus other stakeholders? ). As the categories for the different nodes are not mutually exclusive and studies often included multiple concepts (e.g., fraud and organized crime were reported in the same study), the totals do not equate to the number of included records and instead, vary between nodes.

### How does corruption impact the food system?

In order to assess the impacts of corruption, we focused on studies categorized as providing evidence of impact, rather than descriptive evidence (Table 3). To illustrate the intersections between the type of corruption, the area in which it occurred and its impacts, we developed a Sankey diagram using the approach described above. We also narratively summarized the evidence around each type of impact, citing examples drawn from the included literature.

### What solutions have been proposed to address corruption in the food system?

We narratively summarized the evidence around proposed solutions to corruption in the food system, as presented in the included studies.

**Table 3:** Definitions of concepts utilized in the data synthesis from the included records

Characteristic	Category	Description
Corruption types	Bribery	“The offering, promising, giving, accepting, or soliciting of an advantage as an inducement for an action which is illegal, unethical or a breach of trust. Inducements can take the form of money, gifts, loans, fees, rewards, or other advantages (taxes, services, donations, favors etc.).” [34]
	Fraud	“To cheat. The offence of intentionally deceiving someone to gain an unfair or illegal advantage (financial, political, or otherwise).” [33]
	Corporate political activity	Corporate attempts to shape government policy in ways favorable to the firm [32]
	Bureaucratic corruption	Abuse of one’s position of authority at the bureaucratic and/or political level to benefit a few at the expense of the many. This involves individuals who hold power within a country or organization allowing criminal elements to infiltrate their institutions [33, 42]
	Organized crime	“Organized crime is a continuing criminal enterprise that rationally works to profit from illicit activities that are often in great public demand. Its continuing existence is maintained through corruption of public officials and the use of intimidation, threats, or force to protect its operations.” [35]
Food system actors	Primary and raw material producers	Individuals involved with producing and/or growing raw materials and primary produce, at the early stages of food production. This includes stock and crops, and may contain fruit, vegetables, grain, seafood, farm, or agricultural produce of any description. Examples: farmers, horticulturist, fishermen, farm employees.
	Food processors and packers	Those that handle different types of food produce with the intention to cook, prepare, cut, or package the items. Examples: food manufacturers, butchers, meat packers, daily processors.
	Distributors, transporters, and logistics	Individuals that ensure the transport and storing of food as it travels from producers to food service operators. Examples: food distributors (generally), drivers, truckers.
	Business or corporate actors	Corporate food industry actors or their associates. Examples: food and agricultural corporations, private sector, food and drinks industry, ‘Big food industry’, food industry lobbyists and representatives, corporate/business elites.
	Waste management actors	Individuals and organizations having an interest in food waste management and participating in activities that make that possible. They include enterprises, organizations, households and all others who are engaged in some waste management activity relating to the food system [43].
	Marketers, retailers, traders, and wholesalers	Food service operators who sell food produce or products to consumers. Examples: collectors and auction marketers, wholesaler commercial merchants, retailers, food service, suppliers, traders.
	Government officials and public servants	People who hold a legislative, administrative, or judicial office (either appointed or elected), who work in government or are a public servant [44]. Examples: ‘Public sector’, politicians, politician leaders, state officials, government, public authorities, governing authorities, public servants, local officials, community chiefs/local authorities, government regulators, legislators.
	Public safety and security authorities or regulators	People who hold a legislative, administrative, or judicial office (either appointed or elected) who exercise a public function as part of a public agency. Examples: safety and security authorities/regulators; agricultural or aquacultural regulators, health inspectors, police officers, armed forces, border control, food relief authorities, extension workers.
	Intermediaries	The ‘in-between’ of governance and food production. Examples: landowners; political agents seeking re-election (who will have an interest in keeping water prices low); civil society organizations, development practitioners, academia, investors; water engineers; Local water management institutions; union officials; NGO/NGO donors (*n* = 2); wealthy elites; Cane Society officials; teachers; banks; the environment/ecosystems/wildlife.
	Community members	General members and households in the community who consume food. Examples: community members, subsistence farmers/fishers, households, consumers.
	General food supply chain actors	Some records did not specify the instigators and/or those impacted by corruption and generally referred to ‘food supply chain actors’. This could involve individuals such as primary and raw material producers, food processors and packers, distributors, and transporters, business or corporate actors, or marketers, retailers, traders, and wholesalers.
Food system areas	Policy and governance structures	Systems of policy and governance that ultimately shape food system outcomes and work towards attaining food security for all. These structures interact with the food system in complex and iterative ways, involving both formal and informal rules, norms, and processes [5]. Mechanisms and processes including policies, legislation, planning, finances, monitoring and implementation, that can involve economic, health, social, technological, and environmental sectors, may be in/directly involved-with or impact the food system.
	Systems supporting food production	Ecological, human, energy, economic and health systems that support food supply chains to produce and distribute food [5]. These systems are intermediaries between governance systems and the food supply chain, that influence the functions of the food system, but are not directly situated in the food supply chain, e.g., NGOs or financial bodies that fund agricultural programs, police institutions that regulate food transport, landowners who lease out property to farmers.
	Food supply chains	Includes all stages and actors, including private sector businesses, from production, storage and distribution, processing, packaging, retail and markets, consumption, to waste disposal. The length of a food supply chain and the actors involved, may vary by region [5, 10].
	Food environments	The physical (e.g., stores or markets), socio-cultural, economic, and political surroundings, which influence consumers’ dietary preferences and how they interact with the food system. Food availability, access, affordability, safety, and quality are all part of the food environment [45].
	Individual behaviors and diets	Individual factors, such as socioeconomic status, personal beliefs and decisions, and overall lifestyle characteristics, that affect behaviors including which foods to acquire, prepare and eat, and how a consumer interacts with their food environment. This shapes a consumer’s food choices and diet in terms of quantity, quality, diversity, safety, and adequacy of food. Diets shape outcomes that affect other systems, for example, nutritional impacts within populations that affect health systems [5, 10].
Evidence types	Descriptive	Described corruption in the food system context of interest. Provided insight pertaining to who might be involved with the corruption, what happens, where, when or why it may occur, or how it occurs in the relevant food system context.
	Impact	Investigated or reported on how corruption impacts the food system, e.g., financial loss, environmental damage, decreased agricultural productivity, or how an anti-corruption intervention impacted corruption in the food system context, e.g., testing a technology to measure food fraud, policy, or program to address corruption.
General category	Systemic	Relevant to the categories of ‘food systems areas’ and ‘food system actors’. Indicates that corruption is present in all food system areas or relates to all actors involved. Authors in the included records either explicitly described corruption as being ‘systemic’ in their context or identified numerous areas and/or actors that covered all the defined categories within each domain.

## Results

**Fig. 1:**
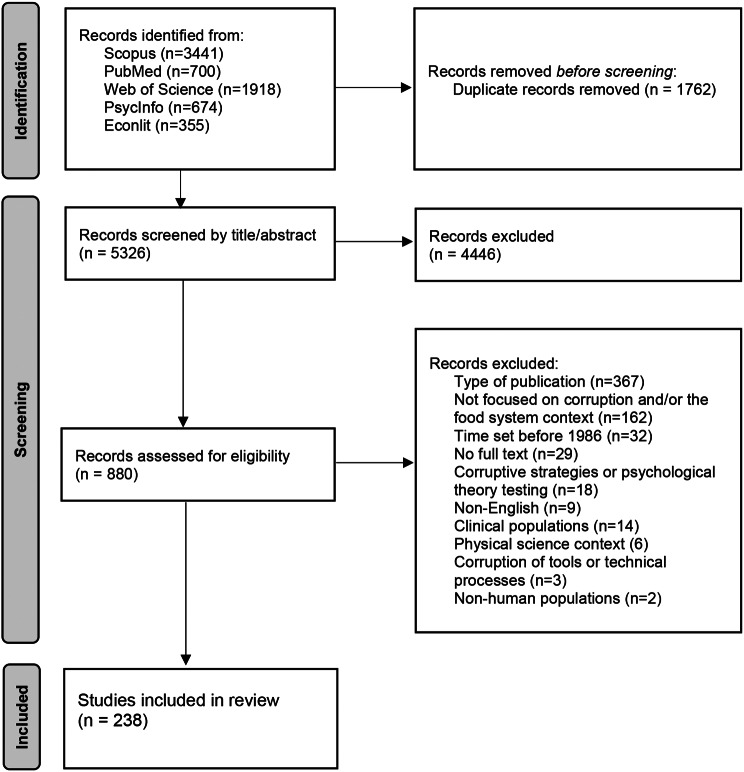
PRISMA flow diagram

Our search identified 5326 records after duplicates were removed. Of these, a total of 238 articles met the inclusion criteria (see Fig. 1).

**Fig. 2:**
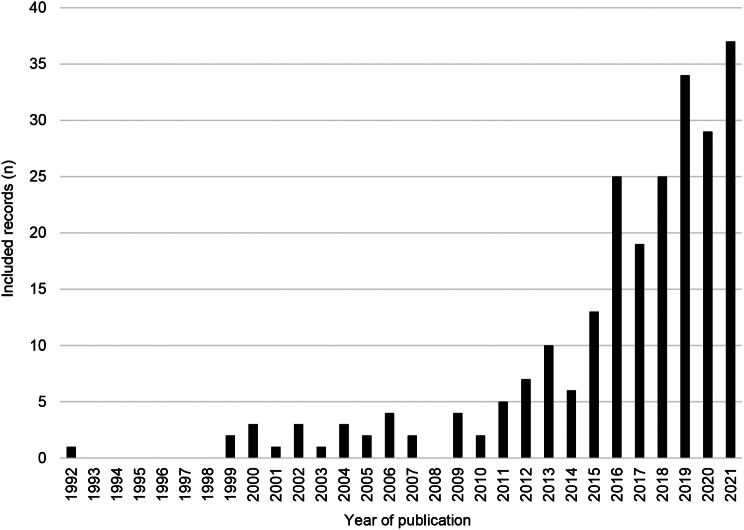
Records included in the scoping review by year of publication (*n* = 238)

Most studies were focused on Sub-Saharan Africa (*n* = 55, of a total of 238 records) and Europe and Central Asia (*n* = 54), followed by East Asia and Pacific (*n* = 37), South Asia (*n* = 25), North America (*n* = 19), Latin America and The Caribbean (*n* = 13), and Middle East and North Africa (*n* = 5). Additionally, 30 papers studied corruption at the global level, including multiple regions. High- (*n* = 68, of a total of 238 records) and lower-middle-income (*n* = 67) countries were most commonly studied. Studies at the global level involving various income brackets (*n* = 48), and those of upper-middle-income (*n* = 39) nations, were also frequently investigated. Low-income nations were the least studied (*n* = 16) from the included literature in this review. Included studies were published between 1992 and 2021. Of the total, almost 90% of the records were published after 2010 (refer to Fig. 2).

A similar number of articles used quantitative (*n* = 101) and qualitative (*n* = 99) study designs. The remaining 38 papers used mixed methods approaches. Studies used many approaches to collecting data on corruption in the food system, and this choice was often informed by the authors’ interpretations of corruption in their food system context. Supplementary File 3 summarizes the methodological approaches taken to capturing corruption. The quantitative approaches to measuring corruption included macro-level analysis, applying standardized internationally comparable indicators such as the Corruption Perception Index developed by Transparency International and the World Bank’s ‘control of corruption’ measure; micro-level analysis, where a proxy variable was developed to represent the specific type of corruption, often at a local or national level; and modelling analysis where empirical data was used to test the predictive power of the model. Qualitative approaches included ethnographic research, case study analysis, content analysis, and interview data collection.

The types of corruption investigated in the food system context were also heterogenous and terminology was used inconsistently. However, it was possible to identify conceptually distinct types of corruption: bureaucratic corruption (*n* = 105), fraud (*n* = 68), organized crime (*n* = 56), corporate political activity (CPA) (*n* = 38), and bribery (*n* = 33). The descriptive characteristics for the included records stratified by corruption type are presented in Table 4 below (full study details in Supplementary File 4).

**Table 4:** Descriptive characteristics of the included records. Ns represent the frequency of concept counts stratified by corruption type

Characteristics	Total, *N* (%)*	Bureaucratic corruption	Fraud	Organized crime	Corporate Political Activity	Bribery
**Overall totals**	300 (100)	105	68	56	38	33
**Country income**	300 (100)					
High	81 (27)	5	34	20	21	1
Upper middle	52 (17)	14	12	12	8	6
Lower middle	95 (32)	48	7	18	2	20
Low	20 (7)	11	1	4	1	3
Multiple	52 (17)	27	14	2	6	3
**Region**	300 (100)					
East Asia and Pacific	47 (16)	11	12	12	8	4
Europe and Central Asia	64 (21)	5	30	16	11	2
Latin America & the Caribbean	17 (6)	5	0	5	5	2
Middle East and North Africa	8 (3)	3	3	0	0	2
North America	21 (7)	0	9	4	8	0
South Asia	38 (13)	21	1	5	0	11
Sub-Saharan Africa	73 (24)	39	6	13	3	12
Multiple countries/global	32 (11)	21	7	1	3	0
**Food system area**	381 (100)					
Policy and governance structures	147 (39)	83	8	21	11	24
Systems supporting food production	65 (17)	24	7	15	6	13
Food supply chains	140 (37)	20	54	28	31	7
Food environments	16 (4)	6	2	5	0	3
Individual behaviors and diets	1 (< 1)	1	0	0	0	0
Systemic	12 (3)	4	2	2	NA	4

*Percentages were calculated based on the total number of concepts for each category and rounded to the nearest whole number

### How is corruption in the food system characterized in the peer-reviewed literature?

We categorized corruption into five types as described in Table 3: bureaucratic corruption, fraud, organized crime, corporate political activity and bribery. Examples of each type of corruption, as well as approaches to capturing and collecting data used in the literature, are summarized below.

### Bureaucratic corruption

Bureaucratic corruption was the type of corruption most frequently identified in the food system context. While it was studied in all country income groups, it was most commonly studied in lower middle income countries (*n* = 48). North America was the only region where bureaucratic corruption was only studied as part of records investigating multiple countries and/or reporting global-level aggregate indicators. Overall, most studies in this category involved the public sector. Political corruption, political influence, rent seeking (i.e. extracting wealth through political or social power), and clientelism (i.e. trading political power for goods and services) were types of bureaucratic corruption specific to the public sector. Public sector corruption was frequently investigated through macro-level indicators (utilizing standardized internationally comparable indicators such as the Corruption Perception Index by Transparency International and World Bank’s governance indicators, namely the ‘control of corruption’ measure) to understand institutional relationships [46–48]. Context-specific explorations of corruption involving governments or state officials were also identified through a range of methodological approaches, including ethnographic studies to understand ambivalent personal relatedness in public office, or case-study analyses involving key informant interviews with those experiencing the bureaucratic corruption [49–52]. The subtypes of patronage, regulatory capture, coercion, nepotism, cronyism, negligence of duty, conflict of interest and extortion generally applied to a range of food system areas and actors [23, 53].

### Fraud

Food fraud was the most common type of fraud studied, involving food industry actors who altered food products in a way that deceived citizens but enabled corporations or businesses to gain profits. Fraud was most commonly studied in high-income nations (*n* = 34). Examples of food fraud include the 2013 horsemeat scandal in the European Union, compromised safety of infant formula in China, and more generally, cases where product authenticity was not upheld (e.g., extra-virgin olive oil, halal meat products, seafood) and resulted in food safety issues for communities [54–57]. The consequences of food fraud on consumer trust in the food industry and farmers’ trust in the authorities and other food system actors were also commonly investigated [58–62]. Other identified types of fraud were agricultural fraud (e.g., contaminated crop pesticides), identity fraud, forgery, financial fraud and theft of public funds, computer fraud, and food stamp fraud [63–67].

### Organized crime

Organized crime was present in the global food system in diverse ways. This included illegal, unreported, and unregulated fishing; labor exploitation of farm or restaurant workers; resource leakage or diversion of funds, particularly in food subsidy or welfare programs; collusion; land grabbing; money laundering using the structures of food production as a pawn; embezzlement; and reoccurring instances of theft or pilferage [20, 59, 68]. A common area of overlap was found between organized crime and fraud, where ‘food crimes’ were described. Examples of these ‘food crimes’ include farmers experiencing repeated exploitation or theft of stock within the meat supply chain, and subsidy leakage and diversion in public distribution programs which particularly affected vulnerable communities [69, 70].

### Corporate political activity

Corporate political activity (CPA) largely concerned acts of lobbying, but also captured any tactics that corporations and businesses used to influence policies that affected the food system (e.g., sugar taxation, agricultural subsidies, obesity prevention legislation) [71–73]. These activities were typically legal in their contexts, and were often seen as a legitimate and accepted part of the democratic process in democratic countries. CPA captures what some study authors call a ‘grey area’ of corruption which, while legal, involves behaviors that influence food governance and policies for the private gains of industry [74, 75].

### Bribery

Examples of bribery involved excess financial payments in exchange for goods, such as food stamp cards, or services. Services provided in exchange for bribes included transporting food products across borders or providing a positive food safety inspection result regardless of whether products or premises met regulatory standards [21, 76, 77]. Bribery was often captured by measuring discrepancies between the expected versus actual cost of a service or item, or through accounts of paying off an individual in a position of authority. In some cases, it was merely stated that ‘bribery’ was present without elaboration. While at other times, bribery was sub-categorized as gift-giving or kickbacks. Gift-giving involved the transfer of resources (that were not necessarily financial) in exchange for a favor. Presented as a sociocultural norm, descriptions of gift-giving were less negative in tone compared to other forms of bribery [21, 78, 79].

### What actors are involved in corruption in the food system?

**Fig. 3:**
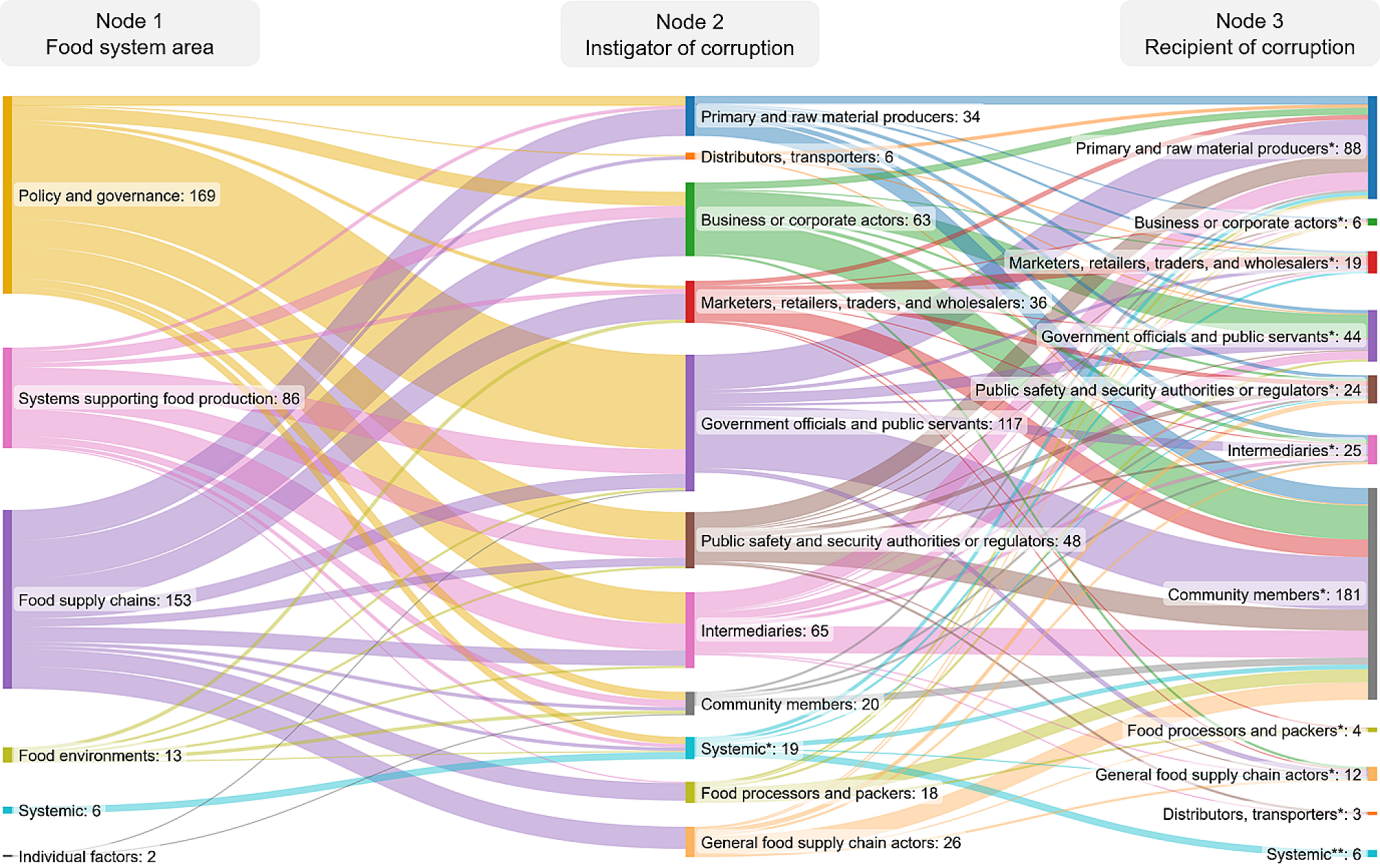
Sankey diagram identifying the flow of corruption among food system actors. The width of each flow is proportional to the total number of concepts identified in the literature for that node, representing a salience of these concepts across the literature base. As the categories for the different nodes are not mutually exclusive the totals vary between nodes. Ns represent the number of concepts identified for each category. (*) Differentiates similar-named categories across different nodes

Figure 3 illustrates the flow of corruption across the food system from actors who are instigators of corruption to those impacted by corruption (see Supplementary File 4 for full details of included studies). Within policy and governance structures, government officials and public servants were the most frequently identified instigators of corruption (*n* = 81), where their behaviors mostly impacted community members (*n* = 45). Intermediaries (*n* = 27) and public safety and security authorities or regulators (*n* = 24) were the next most frequent instigators of corruption within policy and governance structures. Notably, within food supply chains, every category of actor was found to be involved with instigating corruption in this food system area, though business and corporate actors were the most frequent instigators (*n* = 33). While community members were most commonly impacted by corruption, they were rarely identified as the instigators of corruption. In contrast, business or corporate actors were often identified as instigators of corruption but were impacted by corruption on only a few occasions (see Supplementary File 5 for full distribution of instigators and those impacted by corruption).

#### How does corruption impact the food system?

**Fig. 4:**
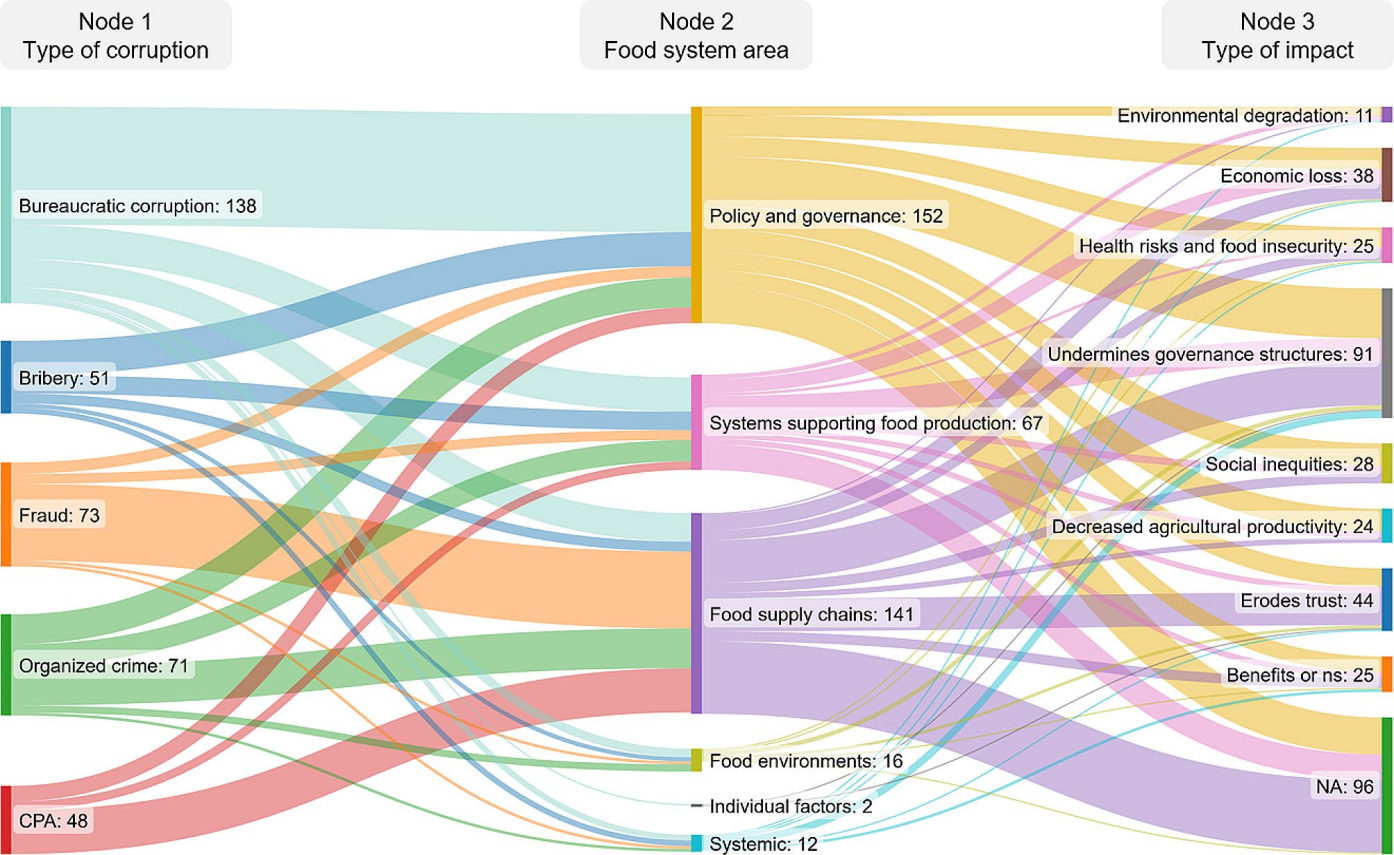
Sankey diagram identifying the flow of corruption across the food system areas and its eventual impacts. The width of each flow is proportional to the total number of concepts identified in the literature for that node, representing a salience of these concepts across the literature base. As the categories for the different nodes are not mutually exclusive the totals vary between nodes. For Node 3, the NA category represents papers that did not report on the impacts of corruption and were classified as ‘descriptive’ studies

Of the included studies, 155 records reported an impact of corruption on the food system. Figure 4 illustrates how corruptions impacts the food system in different ways (see Supplementary File 4 for full study details). The impacts on the food system were primarily negative, though there were also nuances, where corruption was depicted as being interwoven with the food system and a part of some of its functions and mechanisms. A summary of how corruption impacts the food system is described below.

### Undermines governance and regulatory structures

Corruption undermined food system governance structures. Namely, corruption resulted in inefficient operations, impaired accountability, poor performance and lack of transparency. This could have ripple effects beyond the food system, creating barriers to addressing climate change, for example by undermining equitable access to funds and infrastructure made available to support adaptation to climate change [80, 81]. Corruption also undermined food safety: in some cases, failed health inspections were dismissed or food production had decreased input quality (e.g., seeds, chemical fertilizers, and pesticides were below acceptable standards), and surveillance was relaxed to conceal substandard food practices [21, 50, 82, 83].

Officials responsible for governing society often undermined governance systems, using their position of authority as an opportunity for private gain. State officials often sought bribes from individuals working within the food system, normalizing corruption throughout the system [78, 82, 84]. In one study, truck drivers transporting food were threatened with transit delays by police officers unless they offered a bribe [76]. In another example, artisanal fishers found it more beneficial to bribe officials than obtain a formal license for their vessel. While the bribe could cost substantially more than a fishing license, it exempted them from further fishing controls [59]. In these cases, individuals found it necessary to engage in corrupt practices to protect their livelihoods [81].

Corruption also undermined the democratic process and attempts at socioeconomic and political reform. Patron-client relationships were often part of an informal governance system that accorded private sector actors a degree of decision-making power over regulations or public policy. This dynamic was documented in the literature in contexts including Indonesian fishing laws [85], food commodity market prices [83], food welfare subsidies [86], and food inspection regulations [21]. Governance was also undermined by buying votes to influence political outcomes [87], censoring public health campaigns [88, 89], or influencing academics to frame evidence and public opinion in a way that favored industry interests [56].

Similarly, the grey area of corruption involved corporate political activities that, although legal, also interfered with policy and government decision-making processes [72]. For example, actors used strategies including media and public mobilization, lobbying, contributions to election campaigns, or creation of kinship and social ties between business and political elites, to prevent meaningful agricultural or food tax reform that aimed to redistribute costs away from consumers, farmers, or individuals with low socioeconomic backgrounds, to corporations and the rich [90–92]. Food industry lobbying that weakened policy responses to address diet-related disease was also investigated [93]. Shifting the focus of government policy away from socio-structural factors to individual responsibility, and from nutrition to physical activity is an instance of this [94], as well as abolishing the formulation of a sugar tax [95]. Moreover, it was reported that corruption weakened governance but weak governance also allowed corruption to occur, creating a self-perpetuating cycle of more corruptive behaviors [96].

#### Leads to environmental degradation

The presence of corruption was reported to lead to environmental degradation in various forms. This included overexploitation of species and natural resources, such as declining local fish supply and catches due to unregulated fishing, threats to wildlife, disregard for climate change and the environment, and greater deforestation when higher levels of corruption were present [52, 97, 98].

#### Decreases agricultural productivity

Corruption led to decreased agricultural productivity in various ways. It reduced farmer cropland expansion and caused farmers to abandon farmland; limited the number of animals that could be profitably sold due to excess costs of corruption (e.g., due to bribes or informally changed rules and regulations); and affected smallholder farmers’ and traders’ ability to participate in food production due to inflated costs [99, 100]. The consequences of corruption for agricultural productivity are also compounded by resource leakage causing reduced agricultural output for farmers, and reduced labor capacity for farming due to workers migrating away from areas where corruption was inevitable [101–103].

#### Threatens health, safety, and food security

Corruption poses numerous threats to health, both at the individual and national level. Whether it was decreased caloric intake due to high food prices and lack of food accessibility (i.e., from having to pay bribes), or health risks due to the consumption of unsafe food in the case of food fraud, corruption was described as negatively impacting physical and psychological health [56, 104]. When workers were involved, e.g., at a restaurant or farm, corruptive acts involved exploitation that led to consequences to health, safety, and even life [105–107]. At the macro level, decreased national life expectancy, and increased food insecurity, malnutrition, mortality, and armed conflict, were other reported impacts of corruption [104, 108, 109].

### Erodes trust

The erosion of trust within communities was another byproduct of corruption in the food system. Decreased consumer confidence in products linked to corruption negatively impacted purchasing behaviors, food preferences, and perceptions of brand credibility [58, 62, 110]. Moreover, the exposure of corruption within the food system threatened social order and undermined community relationships, as it fueled community doubt in authorities and those in power [56, 111].

### Economic loss

Financial or economic loss due to corruption were also present in various areas of the food system. At the household level, corruption was a financial burden due to overpayments for products and lowered income, especially impacting people in low-income brackets [104, 112]. Furthermore, unequally distributed welfare payments placed further financial pressure on food insecure households. The cost of participating in food production in the presence of corruption, (e.g., paying for land, administrative fees, etc.) caused financial losses for farmers and businesses, resulted in unstable markets, and increased downstream costs in the food supply chain [113, 114]. At the national level, the presence of corruption diverted investors’ financial aid and foreign direct investments, discouraged business activity, and led to loss of output and employment [103, 115–117].

## Widening social inequities

The widening of social inequities was another impact of corruption in the food system. Segregation, racism, and social exclusion were perpetuated by corruption [92, 118]. Whether it was at the shop level where households belonging to ‘lower’ castes were unable to buy products, or at the national level, where villagers were stripped of their land rights to enable lucrative business development, the power imbalance that often complemented corruptive behavior further exacerbated social inequities. Low-income households, minority groups, and smallholder farmers were disproportionately affected [111, 119]. For smallholders in particular, marginalization occurred when large-scale farms captured most of the market due to patronage relations and power imbalances [92, 120, 121]. Moreover, diversion of funding and resources, and market price instability also had greater impacts on smallholders’ participation in food production activities [122, 123].

### Nuance in the impacts of corruption

Despite the negative impacts of corruption in the food system, there was some nuance in the portrayal of corruption in the literature. In some cases, studies highlighted that corruption was tightly interwoven with the food system, and was a key part of some of its functions and mechanisms. Corruption was seen as a mechanism to compensate for bureaucratic failures throughout the food supply chain, and a norm to the functionality of governance systems to progress policymaking [21, 50, 124–126]. In these instances, tackling corruption without looking at its broader context may have unintended consequences. In other cases, corruption itself had positive unintended consequences. Agricultural productivity was negatively impacted by corruption, but this was reported as a benefit for the environment as natural habitats were protected from cropland expansion and deforestation [101, 127, 128]. Positive policy responses to corruption were also reported, where, after corruption was identified, as in the case of food fraud, industry and government were incentivized to be more transparent, introduce better regulatory standards, and address the issues to regain consumer trust [129, 130]. Finally, some studies reported no significant impacts of corruption in their analyses [63, 71, 131, 132].

### What solutions have been proposed to address corruption in the food system?

Few studies focused on potential solutions to address corruption in the food system, while many discussed the critical role of effective governance structures and processes. In terms of empirical research investigating approaches to address corruption, technological solutions were proposed, such as switching to digital food ration cards to prevent resource leakage and using blockchain to address food fraud traceability [133–135]. In line with seeking better approaches to monitoring corruption in the food supply chain, improved predictive modelling methods and global standardization of detecting corruption were also proposed [47, 136]. Finally, an organizational approach to problem solving was explored, where social farming or social enterprises were effective societal organization structures for disempowering organized crime and weakening criminal control [137, 138].

## Discussion

The findings of this study emphasize the complexity of corruption in the global food system. Across the 238 included records, corruption in the food system was studied across a range of country income brackets in the past decade. Five main types of corruption were identified in the literature related to the global food system: bureaucratic corruption, fraud, bribery, organized crime, and corporate political activity. Corruption spanned across various areas of the food system and was commonly observed in policy and governance structures. A total of 155 studies reported on the impacts of corruption on the global food system, with no definitive pathway demonstrating how corruption flowed into eventual impacts. Corruption undermined food system governance and regulatory structures; threatened health, safety, and food security; led or contributed to environmental degradation, economic loss, erosion of trust, and social inequities; and decreased agricultural productivity. The impacts of corruption were nuanced, for example, in some cases corruption led to societal benefits or had no apparent effects on society. A pattern of power imbalances was identified, where community members and primary and raw material producers were disproportionately impacted by corruption, while the instigators were commonly public and private sector actors. Although few solutions were proposed, some were promising in addressing corruption in the food system, such as predictive modelling to improve detection of corruption and organizational approaches to problem solving.

### Insights from findings and comparison with existing literature

Synthesizing the literature to understand corruption in the applied food system context is necessary to recognize the context-dependent variability of corruption [17–19]. To our knowledge, this scoping review was the first to systematically investigate corruption in the global food system. A report describing anti-corruption measures in the agricultural sector found corruption affected all levels within the sector including the input, production, processing and packaging, storage and distribution stages as well as the consumer interface [139]. Although the report does not encompass the whole food system, this report supports the finding in the current review that addressing corruption in the agricultural subsector of the food system is complex [139].

The review identified characteristics of corruption and the diverse ways in which it affects different areas of the food system. The finding that corruption in the food system was not localized to one particular income group, reinforces the inaccuracy of longstanding beliefs that corruption is a “third-world” or “developing country” problem [140–142]. Characterizing corruption in the food system helped to identify ‘legal’ and ‘illegal’ corruption [143], and conclude that corruption is especially present in policy and governance structures and food supply chains. Moreover, the heterogeneity in the approaches to investigating corruption in the food system identified the multidimensional nature of addressing corruption. There were no existing frameworks to guide understanding corruption in food system contexts and individual study findings were dependent on the authors’ conceptualizations of the phenomenon. This demonstrates the need to use interdisciplinary knowledge to cooperatively identify relevant solutions and holistically address corruption in the food system.

Analysis across the stakeholder categories identified a general trend showing an imbalance of power relating to the impacts of corruption. The burdens of corruption are largely being placed on more vulnerable groups, such as community members and primary food producers, while government officials and public servants, intermediaries, and business and corporate actors, are most commonly instigators of corruption (Fig. 3). Moreover, the identified impacts, such as social inequities, economic loss, decreased agricultural productivity, and health risks and food insecurity (among others), also disproportionately affect those with the least amount of power. By illustrating the flow of corruption in the food system (Fig. 4), insights into the connections between the types of corruption, areas of the food system, and the eventual impacts were uncovered. Given the widespread presence of corruption across the food system, working towards more sustainable and equitable food systems should incorporate the effects of corruption, as it may further exacerbate inequities if unaddressed [144, 145].

## Implications for research and practice

Understanding how corruption presents itself in the food system, where it exists, who is involved, and how it flows throughout the food system to its eventual impacts highlights potential areas for intervention that could support the food system transition. Given that the impacts of corruption are largely negative and there is little consideration for corruption in the existing policies and agendas for a food system transition [2, 146], failing to integrate measures to address corruption may undermine efforts toward attaining a healthier, more equitable, food system. The evidence from this review may assist with informing and developing anti-corruption policies and programs. Since there were few studies describing proposed solutions to corruption in the food system, developing, evaluating, and reporting anti-corruption measures within the applied context is necessary [139, 147].

The complex nature of corruption in the global food system, along with the limited number of solutions to address it, present the need for interdisciplinary and multi-sectoral approaches to developing solutions to minimize corruption. Conceptualizing corruption through a systems lens and recognizing the totality of the food system’s components and drivers may help to address the limitations of previous efforts to improve food security and nutrition [4, 13]. A systems-informed holistic lens allows us to unpack the complexity of how corruption impacts social systems and the macro-level collective dynamics in the global food system [144, 145].

Moreover, system theory and explorations of the perspectives relating to corruption have suggested that corruption is deployed as a moral language that shifts according to political-economic and power relations [141]. The nuanced findings from our review identifying that corruption may be interwoven in the functions and mechanisms of social and political systems, and is not bound by geographical regions or income levels, reinforces the complexity of addressing corruption. As corruption often involves a selectively applied and ‘slippery’ discourse [141], the measurement of corruption further confounds our understanding of the phenomenon. Although it might not fit conventional definitions, conceptualizing corruption as a challenge that includes ‘legal’ forms of corruption and that is widespread across the globe, may provide critical insight into unjust practices and issues relating to corruption in the global food system [141, 143, 145, 148].

## Strengths and limitations of the review

The review is strengthened by our use of a broad and neutral definition of corruption to inform our investigation of corruption in the global food system. The broad definition limited bias from existing perceptions of corruption and enabled an inclusive understanding of corruption. The review considered samples from low- to high-income nations across numerous geographical regions, and a wide range of study contexts and corruption types. An iterative deductive and inductive approach was used to guide the review, to maintain an understanding that is adaptive and reflective of corruption in multiple contexts.

The findings of this review are limited to what has been studied in peer-reviewed literature. Therefore, these findings represent the scope and breadth of empirical research, but are likely to exclude other essential scholarship related to defining and characterizing corruption broadly, debates related to the role of commercial entities and governance and corruption that could be applied to the area of global food systems and corruption. Beyond the academic knowledge base, grey literature may contain additional information on this topic. Many cases of corruption in the food system may be hidden and challenging to document: identifying, measuring and studying corruption is challenging and sometimes dangerous. Moreover, findings suggest there are food system areas where corruption has not been studied. For example, although a recent report by the European Commission testified that the waste sector is prone to corruption at the local level, corruption in the waste management sector was not described in the included literature [149]. Although we used data charting templates to allow for consistent reporting throughout the review, the findings of this review are subject to author bias given the nuanced nature of corruption. The scoping review was also limited to English-language articles, potentially missing relevant literature that is outside this scope.

## Conclusion

This systematic scoping review aimed to understand the characteristics, involved actors, impacts, and empirical evidence for approaches to address corruption in the global food system. The findings from this review characterized the types of corruption in the food system and their eventual impacts, identified the actors involved, and synthesized the limited evidence for potential solutions. These findings could support the essential but often overlooked topic of corruption in global governance of food systems and support researchers and policymakers in developing, implementing, and evaluating anti-corruption measures to aid efforts to build an equitable, sustainable, and healthy food system for all.

### Electronic supplementary material

Below is the link to the electronic supplementary material.

Supplementary Material 1

## Data Availability

All data generated in this review is included in the manuscript and supplementary materials. The data source for the review consisted of articles which are available from their respective publishers.
