# Supplementary material for: The role of corruption in global food systems: a systematic scoping review
Source: Global Health. 2024 Jun 15;20:48. doi: 10.1186/s12992-024-01054-8 (PMC11179269; doi:10.1186/s12992-024-01054-8)
Supplement: Supplementary file 1 — Supplementary Material 1 [file 12992_2024_1054_MOESM1_ESM.docx]

# **SUPPLEMENTARY MATERIAL**

*Supplementary file 1. Search strategy*

Population: to search for all possible actors within the food system, no keywords were used to avoid limiting the search.

Concept: corrupt* OR bribe* OR theft OR nepotism OR "organized crime" OR “organised crime” OR lobby* OR crony* OR patronage OR "failed state" OR "legislative capture" OR crime OR “illicit practice” OR dishonest* OR anticorrupt* OR “anti-corrupt” OR antifraud OR “anti-fraud*” OR “informal payment” OR “informal practice” OR fraud* OR quackery OR forgery OR absenteeism OR embezzl* OR collusion

**AND**

Context: food OR "food system*" OR "food insecur*" OR “food secur*” OR "food sovereignty" OR "food trade" OR "food subsidy" OR "food policy" OR malnutrition OR undernutrition OR overnutrition OR agricultur*

**AND NOT**

assay OR cell* OR spectroscopy OR spectrometry OR DNA OR chem* OR gene* OR nuclear OR mouse OR mice OR rat OR enzyme OR polymer* OR chromatography OR histolog* OR electrode* OR neuro* OR neural OR “in vitro” OR molecul* OR pcr

*Supplementary file 2. Data charting form*

| Study characteristics | Authors |
| --- | --- |
|  | Title |
|  | Year |
|  | Country |
|  | Country regions (using World Bank classifications) |
|  | Characterization of corruption (e.g., bribery, fraud, rent seeking) |
|  | Data collection approach (e.g., corruption perception index score, through qualitative accounts, community observation) |
|  | Study topic (e.g., malnutrition, cattle rustling, food safety) |
|  | What is the ‘evidence type’ being reported? (Impact or descriptive) |
|  | What are the key findings of the study specifically relating to corruption in the food system context? |
| Food system area | Where in the food system does corruption originate? (policy and governance systems, systems supporting the food system, food supply chain, food environments, individual-level involved with consumer behaviors) |
| Actors | What stakeholders or sectors are involved with instigating corruption in the food system? (e.g., ministers in government, community leaders, store owners) |
|  | What population is the recipient of corruption in the food system? (e.g., individual purchasing the food product, farmers producing the crops) |
| Impacts | What are the impacts of corruption in the food system? |
| Solutions | What are the potential solutions to corruption in the food system? |
| Other | Notes/other issues |
|  | Key findings and conclusions |
|  | Limitations of article |

*Supplementary file 3. Approaches to collecting data on corruption in the global food system*

Table S3 summarizes the approaches to collection data on corruption in the global food system. Studies often used multiple approaches to collecting data on corruption and the methods in Table S3 were not always mutually exclusive.

Table S3. Approaches to collecting data on corruption in the global food system

| **Approach to measurement** | **Description** | **Example papers** |
| --- | --- | --- |
| **Macro-level quantitative analysis** | Use of macro-level measures of corruption that were summarized in an index at an internationally comparable scale. Common measures included: the Corruption Perception Index by Transparency International; World Bank’s governance indicators, namely the ‘control of corruption’ measure; and inter-regional systems of reporting corruption (e.g., European Union detection system for cases of food fraud). | (49,130,158) |
| **Micro-level quantitative analysis** | Development of proxy variables for corruption based on the authors’ interpretation of the phenomenon in their respective context (generally at a national/local level). Some common measures included: illicit financial flows calculated using the difference between expected and actual payments for the service/items; quantification of perceived corruption from ‘on-the-ground’ stakeholders; national or local collected data on the corruption issue e.g., lobbying cases, fraud detection cases, cases of recorded organized crime rates. | (18,28,40,44,76,82,145,155,167,238,239) |
| **Ethnographic research** | First-hand investigation of the social and environmental context in which the population of interest is situated in. Data was often collected through participant observation, fieldwork, and/or open-ended interviews. At times, secondary data analysis was used to further understand and explain participants’ perspectives, actions, and behaviors. The timeframe of data collection ranged from over numerous months to multiple years. | (62,81,116,129) |
| **Case study analysis** | A deep observation of the characteristics of an individual, a group or a community, in aims to analyze various phenomena relating to the unit of study. Can involve qualitative and/or quantitative data collection, such as surveys, fieldwork, interviews, and content analysis. | (6,33,66,79,92,97,111,166) |
| **Interviews*** | Qualitative interview studies were conducted to explore the relevant type of corruption in the food system context. This included an array of semi-structured, in-depth, and/or focus group interviews. | (67,108,157) |
| **Content analysis** | Analysis of government documents, newspaper articles, media documents, or Twitter posts. | (89,100,119,124,138,141,168) |
| **Modelling analysis** | Models were developed using a data driven approach and validated/tested using empirical data that measured corruption in the food system. | (240) |
| **Tool development** | Tools to predict or screen for instances of corruption were developed based on empirical (quantitative or qualitative) data. | (23,187) |
| *Content analysis, case study analysis, and analyses using an ethnographic approach often involved interviews as part of their studies. | | |

*Supplementary file 4. Study characteristics of all the included records (n=238). The key identifies the codes used to classify the study characteristics for each area of interest.*

| Key | | | | | | | | | | |
| --- | --- | --- | --- | --- | --- | --- | --- | --- | --- | --- |
|  | Corruption type/s**^†^** | | | Food system area/s**^§^** | Stakeholders involved**^β^** | Impact type/s**^α^** | | | | |
| a | Bureaucratic corruption | | | Policy and governance structures | Primary and raw material producers | Undermines governance and regulatory structures | | | | |
| b | Fraud | | | Systems supporting food production | Food processors and packers | Environmental degradation | | | | |
| c | Organized crime | | | Food supply chains | Distributors, transporters, and logistics | Decreased agricultural productivity | | | | |
| d | CPA | | | Food environments | Business or corporate stakeholders | Health risks and food insecurity | | | | |
| e | Bribery | | | Individual behaviors and diets | Marketers, retailers, traders, and wholesalers | Erodes trust | | | | |
| f | - | | | Systemic | Government officials and public servants | Economic loss | | | | |
| g | - | | | - | Public safety and security authorities or regulators | Social inequities | | | | |
| h | - | | | - | Intermediaries | Benefits or ns | | | | |
| i | - | | | - | Community members | NA | | | | |
| j | - | | | - | General food supply chain stakeholders | - | | | | |
| k | - | | | - | Systemic | - | | | | |
| † What type/s of corruption were identified in the studied food system context?  § What food system area/s did corruption originate in the studied context?  Β Who were the stakeholders involved (instigators and recipients) in corruption in the studied context?  Α What were the impacts of corruption identified in the studied food system context?  NA: refers to the studies that did not report the impacts of corruption and were classified as descriptive studies. | | | | | | | | | | |
| Study characteristics | | | | | | | | | | |
| First author | | Year | Study domain | | | | Corruption type/s | Food system area/s | Stakeholders involved | Impact type/s |
| Abdullah (1) | | 2020 | Political risk factors for food security | | | | a | a | f, h, i | d |
| Agnoli (2) | | 2016 | Consumer perceptions of food safety post food fraud scandal | | | | c | c | e, i | e |
| Akerkar (3) | | 2016 | Patronage and resource leakage in the distribution of government social and welfare programs | | | | a | a | h, i | f, g |
| Alguacil-Duarte (4) | | 2020 | Political opportunism for re-election and low agricultural water prices | | | | d | a | h, i | h |
| Ali (5) | | 2016 | Corruption and the size of food and agribusiness firms | | | | a | c | d, j | i |
| Al-Mutairi (6) | | 2019 | Exploration of corruption’s influence on food inspection | | | | a, b, e | a, c | e, g | a, h |
| Angeles (7) | | 1999 | Political entrepreneurship and rent-seeking activities relating to agricultural taxes | | | | a | a | f, i | a |
| Anik (8) | | 2014 | Determinants of experiencing corruption in farming households | | | | a, e | a, b | a, f, g, i | i |
| Anik (9) | | 2013 | Impact of corruption on the food security of rice farming households | | | | e | a, b | a, f, g | d, f |
| Anik (10) | | 2013 | Household-level determinants of corruption and its different forms | | | | a, e | a, b | a, g | i |
| Ariabod (11) | | 2019 | The impact of governance on total agricultural output | | | | a | a | a, f, j | i |
| Ashby (12) | | 2013 | Effects of organized crime on foreign direct investment and business activity | | | | c | a, c | d, f, h, j | f |
| Asiedu (13) | | 2020 | Impact of Official Development Assistance on agricultural fixed capital formation | | | | a | a | f | f |
| Atwood (14) | | 2006 | Fraud in the federal crop insurance program | | | | b | c | a, f | h |
| Azzam (15) | | 2004 | Involvement of collusion in information pooling mandated by Livestock Mandatory Reporting Act | | | | c | c | b | i |
| Badiru (16) | | 2016 | Consumer perceptions-of using online marketing portals for purchasing agricultural produce | | | | b | b | e, i | i |
| Bahn (17) | | 2019 | Effect of institutional environment on food retail transformation | | | | a | a | e, f | a |
| Banerjee (18) | | 2018 | Subsidy leakage in a rice public distribution program | | | | a, c | a | f, g, i | i |
| Banerji (19) | | 2004 | Impact of collusion on grain market prices and efficiency of government procurement | | | | a | c | b, e | a, f |
| Barbier (20) | | 2012 | The linkages between corruption, rural poverty, and tropical land use trends | | | | a | a | f | b |
| Barclay (21) | | 2007 | Governments influencing profitability and sustainability of tuna industries | | | | e | a | f | i |
| Barnard (22) | | 2017 | Understanding what happened in the EU horsemeat scandal | | | | b | c | a, e, i | i |
| Barnett (23) | | 2016 | Impact of EU horsemeat incident on consumers’ attitudes and behaviors | | | | b | c | i, j | e |
| Baudoin (24) | | 2014 | Smallholder farmers’ insights on local and national support to climate change adaptation | | | | a | a | a, f | i |
| Beekman (25) | | 2013 | Corruption in rural agricultural economies | | | | c | a, b | f, h, i | a, c, e |
| Beg (26) | | 2021 | Relationship between tenancy and political success of landlords | | | | a | b | h, i | a |
| Bélair (27) | | 2021 | Politization of land formalization for farmland investments | | | | a | a | f, i | a, g |
| Bellemare (28) | | 2015 | Corporate influences on legislative action on agricultural policy in US Congress | | | | d | a, b | f | a |
| Benbrook (29) | | 2014 | Tracking contamination in crop pesticides | | | | b | NA | i | h |
| Benjaminsen (30) | | 2012 | Influence of climate variability and environmental conditions on land-use conflicts | | | | a, e | a | f, i | a, e |
| Benjaminsen (31) | | 2009 | Factors leading to a farmer-herder conflict | | | | a, e | a, c | a, f, i | a, e |
| Bergeron (32) | | 2019 | Corporate influence on obesity policy | | | | d | b, c | d, f, i | a |
| Beseng (33) | | 2019 | Interrelationship of the different criminal practices in fisheries | | | | a, b, c, e | f | f, j, k | a |
| Bhuyan (34) | | 2000 | Welfare impact of corporate political activities in US food manufacturing industries | | | | d | c | b, f, i | a |
| Bimbo (35) | | 2019 | Simulating fraud related to untruthful Extra Virgin Olive Oil claims | | | | b | c | b, i | e, f |
| Bødker (36) | | 2015 | Political processes that led to the introduction and rapid abolition of the fat tax | | | | d | c | d, f, h, i | a |
| Boerder (37) | | 2018 | Mapping the role of transshipment in transnational fishing industry | | | | c | c | a, e, h | i |
| Bouzembrak (38) | | 2016 | Assessing and modelling food fraud notifications in a Rapid Alert System for Food and Feed | | | | b | c | a, g | i |
| Brock (39) | | 2021 | Corporate agricultural lobbying in US institutions | | | | d | c | d, f | i |
| Bromley (40) | | 2011 | Implications of bribes and transit delays on the movement of agricultural commodities | | | | e | b | a, c, g, h | a, f |
| Brooks (41) | | 2017 | Changes instigated by UK industry and government to address food fraud issues | | | | b | c | d, i | h |
| Brown (42) | | 2019 | Food, beverage, and tobacco industries’ capacity to influence public health policymaking | | | | a | a | f, d, i | a, e |
| Bulte (43) | | 2007 | Farmers trading bribes or political contributions to politicians for farming subsidies | | | | a, d, e | a, c | a, f | a, c |
| Bumblauskas (44) | | 2020 | Using blockchain to move goods through global supply chains | | | | b | c | i, j | i |
| Burlandy (45) | | 2021 | Private commercial sector influence on food and nutrition regulations and policy | | | | a, d | c | d, f, h, i | a |
| Campbell (46) | | 2019 | Precarious labor conditions of Italian temporary migrant workers in Australia | | | | c | c | a, i | i |
| Carey (47) | | 2016 | Stakeholders and elements involved in development of the Australian National Food Plan | | | | d | c | d, f | a |
| Carrero (48) | | 2020 | Analyzing colonization and deforestation trajectories in southern Amazonas | | | | c | b | f, d, i | a, b |
| Chang (49) | | 2015 | The effect of trade liberalization on environmental degradation and the regime-specific relationships involved | | | | a | a | f | a, b |
| Chapsos (50) | | 2018 | Illegal, unreported, and unregulated fishing and transnational organized crime | | | | c | b | d, h, j | i |
| Che (51) | | 2020 | Rural farmers’ insights into the agricultural crisis | | | | a, b | b, c | a, f, g | i |
| Cheng (52) | | 2012 | Food crime offences involved in food production, selling and marketing, and food preparation. | | | | B, c | f | i, k | a, d, e |
| Chilombo (53) | | 2021 | Interplay between large-scale land acquisition deals and land governance institutional structures | | | | a, e | a, b | f, g, i | a |
| Chirwa (54) | | 2002 | Experiences of small farmers in state-sponsored irrigation scheme | | | | a | a | a, f | i |
| Chukwuone (55) | | 2006 | Constraints and strategies towards effective cost-sharing of agricultural technology delivery | | | | a | d | a, g | a, e |
| Cisneros-Montemayor (56) | | 2013 | Illegal, unreported, and unregulated fishing | | | | c | b, c | a, e, f, g, i | i |
| Cissokho (57) | | 2013 | Cost barriers to the transport of agricultural products in West Africa | | | | e | b | c, g | a, f |
| Collins (58) | | 2010 | The opportunities and grey channels that may exist in a free trade environment | | | | d | a, c | k | i |
| Dandurand (59) | | 2019 | The transition from paper to digital food rationing infrastructure and the reconfigured bureaucratic practices | | | | a, c, e | d | g, i | i |
| Davies (60) | | 2019 | Labour exploitation as corporate crime in food production | | | | c | c | d, g, i | i |
| Davies (61) | | 2020 | Harmful labor practices in agricultural and food supply networks | | | | c, d | c | d, g, i | i |
| De Rosa (62) | | 2016 | Role of organized crime in the region of buffalo mozzarella production | | | | c, d | a, c | k | a, d, e |
| Deshingkar (63) | | 2005 | The functioning and shortcomings of the Food-For-Work program | | | | a, e | a | f, g, i | i |
| Dodson (64) | | 2009 | Factors that enable and impede state-level childhood obesity prevention legislation | | | | d | c | d, f, i | i |
| Ebewore (65) | | 2021 | Farmers’ perceptions of infrastructure availability and conditions in rural communities | | | | a | a, b | a, f | f |
| Ekbom (66) | | 2001 | The conditions that sustainable national development based on agriculture would be attainable | | | | a | a | a, f | a, c, f, e |
| Elsen (67) | | 2021 | Social farming as a tool for developing marginal agricultural areas characterized by a strong presence of illegality | | | | c | a | a, h | i |
| Evans (68) | | 2015 | Evaluate the degree, extensity, and gravity of the consequences of corruption on Nigeria | | | | a | a | f, g, i | d |
| Fazzi (69) | | 2020 | Potential of social enterprises as driving forces for legal and eco-social development | | | | c | a | a, h | i |
| Fearnley (70) | | 2021 | Perceptions of food safety, production, and authentication following food fraud scandals | | | | b | c | a, i | e, h |
| Fikselová (71) | | 2020 | Food fraud in the EU and the inter-country relationships | | | | b | c | j, i | i |
| Fraser (72) | | 2021 | Analysis of corporate power in Ireland’s food and drinks industry | | | | d | c | d, f, i | i |
| Friggeri (73) | | 2021 | Analysis of mafia capitalist accumulation from the vantage point of agriculture | | | | c | b | g, h, i | i |
| Gafițianu (74) | | 2020 | Consumers’ awareness concerning food integrity | | | | b, c | c | j, i | i |
| Galinato (75) | | 2013 | Effect of corruption control and political stability on forest cover through agricultural expansion and road building | | | | a | a, b | a, f | c, h |
| Gharib (76) | | 2021 | Effect of tampering and mishandling on farmers’ willingness to pay for hybrid seed in bags | | | | b | c | a, j | c, e |
| Gluszek (77) | | 2021 | Role of corruption in wild meat supply chains | | | | c | a | k | i |
| Gomez (78) | | 2019 | Effect of socioeconomic factors on access to improved water sources in the rural areas of developing countries | | | | a | a | f, i | h |
| Goodall (79) | | 2021 | Food crimes of the countryside and the rural meat industry collaborators who accomplish them | | | | b, c | c | k | a, d |
| Graham (80) | | 2016 | Role of social protection and food parcels in voting behavior | | | | a, e | a | f, i | i |
| Grydehoj (81) | | 2016 | The political impact of technology in informal governance systems by considering fisheries governance | | | | a | f | k | a, b, h |
| Guntzburger (82) | | 2020 | Food industry perceptions and actions towards food fraud | | | | b | c | f, i, j | i |
| Gupta (83) | | 2014 | Analysis of multi-agency partnership in a participatory watershed development project | | | | a, c | b | f, h | a, f, h |
| Gylfason (84) | | 2000 | Effect of natural resource abundance and extensive agriculture on economic growth globally | | | | a | a | f, i | c |
| Hanf (85) | | 2011 | Impacts of corruption on global children mortality rate | | | | a | a | f, i | d |
| Han (86) | | 2019 | Impacts of food safety risk perception on the different dimensions of governmental trust | | | | a | a | f, i | i |
| Hausermann (87) | | 2018 | Spatial and socio-environmental dimensions of ‘‘small-scale” gold mining | | | | c | a, b | a, i, k | i |
| Hernandez-Aguado (88) | | 2018 | Interactions between public administration, civil society and private companies that could influence health policies | | | | d | c | d, f, g | i |
| Hobbs (89) | | 2004 | The process of federal school meals policy making from 1992 to 1996 | | | | d | c | d, f, h | i |
| Hoff (90) | | 2016 | Reassessing the balance of interests in the EU food information labelling case | | | | d | NA | NA | a |
| Holden (91) | | 2013 | The extent of leakages in a targeted fertilizer and seed subsidy program | | | | a, c | a | f, i | a, g |
| Hossain (92) | | 2014 | The politics of provisions during contemporary food price spikes | | | | a, d | a, c | e, f, h, i | a, d, g |
| Hunt (93) | | 2021 | Ultra-processed food industry’s use of Twitter to influence food and health policy debates | | | | d | a, b | a, f, h | i |
| Hu (94) | | 2017 | The development processes, causes, and impacts of the agricultural cooperatives | | | | b | c | d, f | i |
| Ibrahim (95) | | 2016 | Adopting community cattle ranches and radio frequency identification as strategies for containing cattle rustling | | | | c | c | a, h | f |
| Ingram (96) | | 2017 | Challenges to governing sustainable forest food | | | | a, e | f | e, k | f |
| Isaacs (97) | | 2019 | Low-level poaching and organized crime in the small-scale fisheries sector | | | | a, c | a | a, f, h, i | i |
| Ismalia (98) | | 2021 | Exploring relative deprivation theory in the rice industry | | | | a | a, b | a, f, h | h |
| Jacoby (99) | | 2021 | Governance reform in the Indus Basin watershed canal irrigation system | | | | a | a, b | a, f, h | a |
| Jaichuen (100) | | 2018 | Corporate Political Activity of major food companies in Thailand which relate to obesity and NCDs | | | | a, d | c | d, f, i | a |
| Jeffrey (101) | | 2002 | Insights into low-level economic corruption within institutions responsible for purchasing sugarcane in rural India | | | | a, e | a, b, c | a, f, h | a, f, g |
| Kaitibie (102) | | 2017 | The impact of corruption on Qatari food imports and associated costs | | | | a | a | f, j | f |
| Kalaora (103) | | 2011 | The effect of farmland evictions due to corruption on the white farming community | | | | a | a | a, i | g |
| Kansanga (104) | | 2019 | Threats to agrarian livelihoods from displacement and exploitation of smallholder farmers | | | | a | b | a, k | c, f |
| Kantel (105) | | 2019 | Socio-political narratives around fisheries governance | | | | a | a, b, c | f, g, i | a, g |
| Kassem (106) | | 2020 | Farmers’ risk perception regarding fraudulent pesticides | | | | b | c | a, c, d | e |
| Kassem (106) | | 2021 | Farmers’ recognition and purchasing behaviors regarding fraudulent pesticides | | | | b | c | a, e | e |
| Kaul (107) | | 2018 | Household responses to an inefficient public distribution food subsidy program | | | | a | c | a, c, f | a, c, d |
| Kendall (108) | | 2019 | Chinese consumers’ perceptions of food fraud | | | | b | c | a, b, e, i | d, e |
| Kendall (109) | | 2018 | Food fraud and the perceived integrity of European food imports into China | | | | b | c | a, b, e, i | e |
| Kimanthi (110) | | 2018 | The anomaly of the millennium villages project fixing food and markets in Kenya | | | | a | a | a, c | a, g |
| Kopytko (111) | | 2016 | Barriers to climate change capacity building, adaptation, and mitigation in Ukraine’s agri-food sector | | | | e | a, b | a, i, k | a, b, e |
| Koubová (112) | | 2018 | Food fraud detection in retail markets | | | | b | c | i, j | i |
| Kowalska (113) | | 2018 | Understandings of food fraud among students | | | | b | c | a, i | e |
| Kroetz (114) | | 2020 | The enabling conditions for seafood mislabeling | | | | b | a | f, j | b |
| Kumar (115) | | 2012 | Corrupt practices in public distribution system | | | | a, e | a, b, d | e, f, h, i | f |
| Lammer (116) | | 2017 | Corruption, Transparency, and the puzzle of personal relatedness in a Chinese food network | | | | a | a, d, e | f, i | a, e |
| Lander (117) | | 2017 | Adaptive strategies of smaller foreign investors in the Russian agricultural sector | | | | a, e | b, c | a, d, g | i |
| Larmour (118) | | 2010 | Anti-corruption in Fiji’s post-coup politics | | | | a, e | a | f, g, i | a, e, f |
| Lauber (119) | | 2021 | Attempts of ultra-processed food industry actors in influencing NCD policy at WHO | | | | d | c | d, f, i | i |
| Lee (120) | | 2021 | Prevalence and correlates of food fraud | | | | b | c | e, i | i |
| Levy (121) | | 2020 | Cross-cultural and gender differences in attitudes toward food fraud | | | | b | c | i, j | e |
| Lio (122) | | 2009 | Governance and Agricultural Production Efficiency | | | | a | a | a, f | c |
| Liu (123) | | 2016 | Longitudinal analysis of rice industry food labeling | | | | b | c | b, i | e |
| Liu (124) | | 2015 | Causes of food safety incidents | | | | b | c | b, i | i |
| Lord (125) | | 2017 | Factors and conditions that shape the organization of food fraud | | | | b | c | i, j | i |
| Lovett (126) | | 2017 | Investigation of electronic benefits transfer card reforms in California’s Food Stamp Program | | | | b, c | d | h, i | d |
| Mangla (127) | | 2021 | Food fraud traceability using blockchain technologies in milk supply chains | | | | b | c | a, i | i |
| Mariath (128) | | 2021 | Strategies, practices, and arguments used by the industry to lobby legislators against sugary drinks taxation | | | | d | b | d, f, i | i |
| Martinez (129) | | 2018 | Impacts of manipulation tactics used by bakers to influence laws and regulations relating to business practices | | | | a, e | f | e, i, k | a, h |
| Marvin (130) | | 2016 | Using data-informed Bayesian Network modelling to understand main factors influencing food fraud | | | | b | c | i | i |
| McElwee (131) | | 2017 | Illegal activities in halal sheep supply chain | | | | b, c | b, c | a, b, f, h | i |
| Meenakshi (132) | | 2005 | Collusion and government intervention in paddy auction markets | | | | c | a | e, f | i |
| Mehta (133) | | 2012 | Institutional safeguards against theft in opaque food subsidy programs | | | | c | d | i | i |
| Mehta (134) | | 2014 | Theft of subsidized food and its relation to program ‘opacity’ | | | | c | b | h, i | i |
| Menon (135) | | 2017 | Aadhaar-based biometric authentication in fair price shops to address fraud | | | | b, c | d | e, i | i |
| Mensah (136) | | 2021 | Constraints affecting the poultry farmer’s decision to pay agricultural tax | | | | a | a | a, f | f |
| Mialon (137) | | 2021 | Food industry use of political practices during the adoption of nutrition warning labels | | | | d | c | f, i, j | i |
| Mialon (138) | | 2020 | Corporate political activity of the food industry in Colombia | | | | d | c | d, f, i | a |
| Mialon (139) | | 2018 | Corporate political activity of major food industry actors in France | | | | d | c | d, f, i | a |
| Mialon (140) | | 2017 | Extent to which the dairy industry uses corporate political activity strategies | | | | d | c | d, f, i | i |
| Mialon (141) | | 2017 | Insight into the corporate political activity of the Australian food industry | | | | d | c | d, f, i | a |
| Mialon (142) | | 2016 | Corporate political activity strategies and practices of major food industry actors in Fiji | | | | d | c | d, f, i | a |
| Montgomery (143) | | 2002 | Deregulation of Indonesia’s interregional agricultural trade | | | | a, c | a, b | a, f, g | c, f |
| Moreira (144) | | 2021 | Consumers’ knowledge about food labeling and food fraud | | | | b | c | b, i | e |
| Mulligan (145) | | 2021 | Quantifying interactions related to Bill S-228 and children’s marketing by different stakeholders with the federal government | | | | d | c | d, f, i | i |
| Nading (146) | | 2017 | Explores how food-safety inspectors monitor and certify food service workers and facilities | | | | a, e | a, d | k | h |
| Nagavarapu (147) | | 2016 | Informal monitoring and enforcement mechanisms in public service delivery | | | | a, c | d | e, i | a |
| Naqvi (148) | | 2018 | The role of the financial sector in Pakistan and its effect on productive sector lending to industry and agriculture | | | | a | a, b | f, h | f |
| Neumann (149) | | 2011 | Exploring global irrigation patterns and the relationship with corruption | | | | a | a | a, f, ,i | i |
| Ng (150) | | 2021 | Barriers and facilitators during the policy process of mandatory nutrition labelling | | | | d | c | d, f, i | a, h |
| Ogunniyi (151) | | 2020 | The growth effects of remittances and quality of governance on food and nutrition security | | | | a | a | f, i | h |
| Oliva (152) | | 2021 | The link among money laundering, mafia, and food activities | | | | c | a | e, h, i | i |
| öNDER (153) | | 2021 | The corrupt policies negatively influencing food security in a macroeconomic framework | | | | a | a | f, i | d |
| Osabohien (154) | | 2019 | Using an institutional framework to understand how to improve agricultural sector performance | | | | a | a | f, i | d |
| Pailler (155) | | 2018 | Re-election incentives and deforestation cycles in the Brazilian Amazon | | | | a | a | f, i | b |
| Panigrati (156) | | 2018 | Evaluation of securing food through the public distribution system in India | | | | e | a | g, i | a, g |
| Parkinson (157) | | 2009 | Farmers’ modes of influence within a national agricultural advisory program | | | | d | a, c | a, f, g, h | a, h |
| Pavez (158) | | 2019 | Institutional factors that influence the choice of inter-firm contracts for Chilean apple exports | | | | a | a | f, i | a, h |
| Perone (159) | | 2020 | The relationship between Eco crimes and consumer food and non-alcoholic drinks price index | | | | c | a | d, h, i | i |
| Pittman (160) | | 2019 | Changes in the magnitude and structural patterns of Official Development Assistance funding for fisheries over time | | | | a, d | a, c | e, f, d, i | i |
| Prishchepov (161) | | 2021 | The behavioral intentions of farmers regarding the recultivation of abandoned lands | | | | a | a | a, f | c |
| Quandt (162) | | 2019 | The experiences of child farmworkers in North Carolina | | | | c | a, c | a, h, i | i |
| Raifu (163) | | 2020 | The role of institutions in financial development and agricultural performance | | | | a | a | f, i | i |
| Rezazade (164) | | 2021 | Analyzing food fraud vulnerability factors using a Bayesian Network approach | | | | b | c | i, j | i |
| Riley (165) | | 2018 | Lived experiences of food insecurity in informal settlements in Malawi and exploring the Cashgate corruption scandal | | | | a | a | f, h, i | d |
| Ringsberg (166) | | 2015 | Implementing global traceability standards in fresh Food Supply Chains | | | | b | c | a, c, e, i | i |
| Ritten (167) | | 2019 | Impacts of laundered honey on consumer purchasing behaviors | | | | b, c | c | b, i | e |
| Rizzuti (168) | | 2021 | Involvement of organized crime and mafia-type actors in the food sector | | | | b, c | a, c | d, h, i | i |
| Robertson (169) | | 2019 | The revolving door between government and the alcohol, food and gambling industries in Australia | | | | d | a, c | f, h, i | a |
| Robson (170) | | 2020 | Understanding beef supply chain vulnerability to prevent fraud | | | | b | c | a, b, i | i |
| Rocchi (171) | | 2020 | Evaluation of the socioeconomic impact of food fraud | | | | b | c | g, h, i, j | a, f |
| Ruslan (172) | | 2018 | Muslim consumers’ awareness and perceptions towards halal food fraud | | | | b | c | a, b, i | e |
| Sam (173) | | 2017 | Contaminated stakeholder land management in Nigeria | | | | d, e | b | d, g, h, i | i |
| Schaefer (174) | | 2018 | The effect of the Horsemeat Scandal on European food retailers’ efforts to mitigate fraud | | | | b | c | e | a, e, f |
| Sebhatu (175) | | 2020 | Relationships between membership size and the occurrence of conflict, fraud, and distrust within agricultural cooperatives | | | | b | b | a, g | e |
| Shareef (176) | | 2019 | Complexities surrounding the supply chain logistics for perishable commodities | | | | a | c | e, i, j | a |
| Sheingate (177) | | 2017 | Post-exceptionalism and corporate interests in US agricultural policy | | | | d | b, c | a, d | i |
| Shinn (178) | | 2012 | “General corruption” post-conflict scenarios and the means of resolving agricultural problems | | | | a | a | f, i | c, e |
| Shonhe (179) | | 2020 | Influence of corrupt institutions on medium-scale commercial agriculture | | | | a | a | a, f, g | g |
| Silvis (180) | | 2017 | Fraud vulnerabilities of various actors in the spices supply chain | | | | b | c | i, j | i |
| Singh (181) | | 2021 | Food insecurity during COVID-19 pandemic | | | | a | a | f, g, i | d, g |
| Slangen (182) | | 2003 | Factors determining trust among agricultural stakeholders | | | | a | a | a, f, h | e |
| Smith (183) | | 2000 | The forms and incidence of corruption in agricultural markets | | | | a, e | a | d, g | f, h |
| Smith (184) | | 2017 | Livestock theft and its links to food fraud from a supply chain perspective | | | | c | c | a, h | f |
| Soon (185) | | 2019 | Use of different types of anti-fraud tools within the UK food industry | | | | b | c | b, i, j | i |
| Soyer (186) | | 2018 | The extent to which the availability of liability insurance contributes to illegal, unregulated, and unreported fishing | | | | c | c | a, h | i |
| Spink (187) | | 2016 | Developing a Food Fraud Initial Screening model for risk assessment | | | | b | c | e, d, i | d, e, f |
| Spink (188) | | 2019 | Global perspectives of food fraud | | | | b | NA | g, h | i |
| Spink (189) | | 2019 | International Survey of Food Fraud | | | | b | NA | g, h | i |
| Stamatis (190) | | 2015 | Single tracing method across foodstuffs of animal origin | | | | b | c | b, d, i | i |
| Standing (191) | | 2015 | Criminogenic relationships between corporate and state actors in fisheries | | | | a, c, d, e | a, b | a, d, f | a, b, g |
| Stanikzai (192) | | 2021 | Vulnerabilities of farmers in the production of wheat crop in a war zone | | | | a, e | a | a, g, i | c, f |
| Sugden (193) | | 1999 | Farm crimes in England | | | | b, c | b | a, i | i |
| Sulle (194) | | 2017 | Impacts of class dynamics and social differentiation on sugar cane out growers | | | | a, c | c | a | g |
| Sullivan (195) | | 2013 | Identity, territory, and land grabbing conflict | | | | c | c | i | i |
| Sundstrom (196) | | 2015 | Insights into how corruption hampers law enforcement in the governance of common-pool resources | | | | e | f | k | a |
| Sunge (197) | | 2019 | Impact of agricultural trade liberalization on technical efficiency | | | | a | a | NA | a, c |
| Tagliarino (198) | | 2018 | Expropriation and compensation procedures for farmland in Nigeria | | | | b, c | a | a, f, i | i |
| Tähkäpää (199) | | 2015 | Patterns of food frauds and adulterations reported in the EU rapid alert system | | | | b | c | e, i | d, e, f |
| Thomas (200) | | 1992 | Computer fraud perpetrated against small independent food retailers | | | | b | c | e | i |
| Tibugari (201) | | 2019 | Collusion in the pricing of hybrid maize seed | | | | c | c | a, d | f, g |
| Tracy (202) | | 2016 | Transparency and Food Safety in China | | | | b | a | d, f, i | d |
| Traikova (203) | | 2018 | Motivation behind the intentions of Bulgarian agriculture students to emigrate to Germany for high-skilled farm work | | | | a | a | a, i | c |
| Tse (204) | | 2016 | Insight from the EU horsemeat scandal | | | | b | c | i, j | e |
| Tselengidis (205) | | 2019 | Lobbying arguments and tactics of stakeholders in the food and drink industries relating to the sugar tax | | | | d | c | d, i | a |
| Uchendu (206) | | 2015 | Influence of corruption on food security | | | | a | a | f, i | d, g |
| Uchendu (207) | | 2018 | Hunger influenced life expectancy in war-torn countries | | | | a | a | f, i | d |
| Ujunwa (208) | | 2019 | Impacts of Corruption to Agricultural Export Potential | | | | a | a, b | a, f, g, i | d |
| Uzel (209) | | 2021 | Mutual interaction between corruption and agricultural export variations | | | | a | a | f, h | a, c |
| Vaidya (210) | | 2019 | The interlude between corruption and re-corruption in the context of agricultural marketing | | | | a, e | a | a, f, g | a |
| Van Hoi (211) | | 2013 | Successes and failures of Vietnamese state authorities in regulating pesticides for agricultural purposes | | | | e | a, c | a, d, f | c, g |
| Van Rijswijk (212) | | 2012 | Consumer needs and requirements for food and ingredient traceability information | | | | b | a, c | d, f, i | e |
| van Ruth (213) | | 2020 | Fraud vulnerability in the food service industry | | | | b | c | e | i |
| van Ruth (214) | | 2021 | Company characteristics that make a food business at risk for food fraud | | | | b | c | j | i |
| van Velden (215) | | 2020 | Perceived fairness of bushmeat hunting and consumption regulations | | | | c | c | a, h | i |
| Vandenbrink (216) | | 2020 | Strategies used by industry to influence food and nutrition policy in Canada | | | | d | a | g, h, j | a |
| Venot (217) | | 2011 | Planning and corrupting water resources development | | | | a, e | a | f, h, i | c, g |
| Venter (218) | | 2016 | Change in the global terrestrial human footprint and implications for biodiversity conservation | | | | a | a | h, i | i |
| Vercillo (219) | | 2019 | Farmer resistance to agriculture commercialization | | | | a | a, b, c | a, d, f, g | a, e, g |
| Verter (220) | | 2019 | Dynamics of agri-food trade and food security indicators | | | | a | a | i | i |
| Vollrath (221) | | 2006 | Forces underlying export supply and import demand of two types of food | | | | a | a | NA | h |
| Weber (222) | | 2007 | Relationship between market structure, collusion, and beef and pork retail pricing behavior | | | | a, c | c | e, i | a |
| Weesie (223) | | 2018 | Changes in bonding social capital between the pre- and the post-intervention of water access cooperative structures | | | | a | b | h, i | a, g |
| Wisniewski (224) | | 2019 | Status of food fraud control in Germany | | | | b | c | e, g | i |
| Wolfersberger (225) | | 2015 | Forest transition and land-use change in developing countries | | | | a | a | f, h | h |
| Yami (226) | | 2019 | Influence of power in perpetuating prevailing narratives around public participation in agricultural policymaking processes | | | | a | a | a, f, g, h | a |
| Yan (227) | | 2020 | Perceived fraud vulnerability of the extra-virgin olive oil supply chain | | | | b | c | b, e | i |
| Yang (228) | | 2020 | Vulnerable points in the Chinese milk supply chain | | | | b | c | a, b | i |
| Yankson (229) | | 2016 | Collusion and other constraints faced by farmers in the marketing of their produce | | | | c | c | a, e | i |
| Yasuda (230) | | 2015 | Food safety failures in China | | | | a, c | a, b, c | a, f, g, i | h |
| Yee (231) | | 2019 | Food safety reform and centralizing food safety-related regulatory functions in China | | | | b, c, e | a, b, c | g, i | a |
| Yengoh (232) | | 2016 | Factors that make communities vulnerable to an unequal engagement with large-scale land-investing interests | | | | a | a, b | d, f, g, h, i | i |
| Yu (233) | | 2016 | Governance of the irrigation commons under integrated water resources management in China | | | | a, c | b, c | g, h, i | i |
| Yunusa (234) | | 2018 | The nexus between population, water resources and Global Food Security | | | | a | a | f, i | d |
| Zhang (235) | | 2016 | Economically motivated food fraud and adulteration | | | | b | c | g, i, j | i |
| Zhang (236) | | 2021 | Global hotspots of agricultural conversion risk from multiple crop expansion | | | | a | a | f, g | c, h |
| Zhuang (237) | | 2021 | Quantifying eco-environmental damage caused by illegal fishing | | | | c | c | h, j | b |

*Supplementary file 5. Distribution of stakeholders who were instigators and recipients of corruption based on concept counts.*

|  | Instigators of corruption | | Recipients of corruption | | Ratio |
| --- | --- | --- | --- | --- | --- |
| Stakeholder | N | % | N | % | Recipients/instigators |
| Primary and raw material producers | 27 | 8.2 | 57 | 18.0 | 2.11 |
| Food processors and packers | 15 | 4.6 | 3 | 0.9 | 0.20 |
| Distributors, transporters | 4 | 1.2 | 2 | 0.6 | 0.50 |
| Business and corporate stakeholders | 43 | 13.1 | 6 | 1.9 | **0.14** |
| Markets/retailers/traders | 27 | 8.2 | 15 | 4.7 | 0.56 |
| Waste management | 0 | 0.0 | 0 | 0.0 | NA |
| Public officials (government/public servants) | 84 | 25.6 | 41 | 12.9 | 0.49 |
| Public officials (safety and security authorities; regulators) | 34 | 10.4 | 17 | 5.4 | 0.50 |
| Intermediaries | 42 | 12.8 | 21 | 6.6 | 0.50 |
| Community members | 11 | 3.4 | 133 | 42.0 | **12.09** |
| Food supply chain stakeholders (generally) | 19 | 5.8 | 10 | 3.2 | 0.53 |
| Systemic | 12 | 3.7 | 6 | 1.9 | 0.50 |
| NA | 10 | 3.0 | 6 | 1.9 | 0.60 |
| Total | 328 |  | 317 |  |  |

References

1. Abdullah, Qingshi W, Awan MA, Ashraf J. The impact of political risk and institutions on food security. Current Research in Nutrition and Food Science [Internet]. 2020;8(3):924–41. Available from: https://www.scopus.com/inward/record.uri?eid=2-s2.0-85099806567&doi=10.12944%2fCRNFSJ.8.3.21&partnerID=40&md5=fbe940b7251da915e3fd8a8224c8dfab

2. Agnoli L, Capitello R, De Salvo M, Longo A, Boeri M. Food fraud and consumers’ choices in the wake of the horsemeat scandal. British Food Journal [Internet]. 2016;118(8):1898–913. Available from: https://www.scopus.com/inward/record.uri?eid=2-s2.0-84979272963&doi=10.1108%2fBFJ-04-2016-0176&partnerID=40&md5=67e3cc05aec1ba14ff32f920542b851f

3. Akerkar S, Joshi PC, Fordham M. Cultures of Entitlement and Social Protection: Evidence from Flood Prone Bahraich, Uttar Pradesh, India. World Development [Internet]. 2016;86:46–58. Available from: https://search-proquest-com.ezproxy.library.yorku.ca/ibss/docview/1835027871/249F57A5E765425DPQ/10?accountid=15182

4. Alguacil-Duarte F, González-Gómez F, Del Saz-Salazar S. Urban water pricing and private interests’ lobbying in small rural communities. Water (Switzerland) [Internet]. 2020;12(12). Available from: https://www.scopus.com/inward/record.uri?eid=2-s2.0-85098150508&doi=10.3390%2fw12123509&partnerID=40&md5=1c098fabd141c9892b55bc9950bacdb8

5. Ali J. Performance of small and medium-sized food and agribusiness enterprises: Evidence from indian firms. International Food and Agribusiness Management Review. 2016;19(4):53–64.

6. Al-Mutairi S, Connerton I, Dingwall R. Understanding “corruption” in regulatory agencies: The case of food inspection in Saudi Arabia. Regulation and Governance. 2019;13(4):507–19.

7. Angeles LC. The political dimension in the agrarian question: Strategies of resilience and political entrepreneurship of agrarian elite families in a Philippine province. Rural Sociology [Internet]. 1999;64(4):667–92. Available from: https://www.scopus.com/inward/record.uri?eid=2-s2.0-0033400514&doi=10.1111%2fj.1549-0831.1999.tb00383.x&partnerID=40&md5=551a117b1759dad22e6862e2788b37b7

8. Anik AR, Bauer S. Household income and relationships with different power entities as determinants of corruption. Contemporary Economics [Internet]. 2014;8(3):275–88. Available from: https://www.scopus.com/inward/record.uri?eid=2-s2.0-84907684449&doi=10.5709%2fce.1897-9254.145&partnerID=40&md5=90b85ddad2185b32a0d1e963d6ff3d10

9. Anik AR, Manjunatha AV, Bauer S. Impact of farm level corruption on the food security of households in Bangladesh. FOOD SECURITY. 2013;5(4):565–74.

10. Anik AR, Bauer S, Alam MJ. Why farm households have differences in corruption experiences? Evidences from Bangladesh. Agricultural Economics (Czech Republic). 2013;59(10):478–88.

11. Ariabod A, Moghaddasi R, Zeraatkish Y, Nejad AM. Governance and agricultural growth: Evidence from selected developing countries. ECONOMIC JOURNAL OF EMERGING MARKETS. 2019;11(1):73–80.

12. Ashby NJ, Ramos MA. Foreign direct investment and industry response to organized crime: The Mexican case. EUROPEAN JOURNAL OF POLITICAL ECONOMY. 2013;30:80–91.

13. Asiedu E, Sadekla SS, Bokpin GA. Aid to Africa’s agriculture towards building physical capital: Empirical evidence and implications for post-COVID-19 food insecurity. World Development Perspectives [Internet]. 2020;20. Available from: https://www.scopus.com/inward/record.uri?eid=2-s2.0-85092170404&doi=10.1016%2fj.wdp.2020.100269&partnerID=40&md5=dba85c7c325fa7e47a4c625e141025b7

14. Atwood JA, Robison-Cox JF, Shaik S. Estimating the prevalence and cost of yield-switching fraud in the federal crop insurance program. American Journal of Agricultural Economics [Internet]. 2006;88(2):365–81. Available from: https://www.scopus.com/inward/record.uri?eid=2-s2.0-33645749245&doi=10.1111%2fj.1467-8276.2006.00864.x&partnerID=40&md5=9bed545bbe90b07a880261d489b8b411

15. Azzam AM, Salvador S. Information pooling and collusion: An empirical analysis. Information Economics and Policy [Internet]. 2004;16(2):275–86. Available from: https://www.scopus.com/inward/record.uri?eid=2-s2.0-2642517841&doi=10.1016%2fj.infoecopol.2003.10.003&partnerID=40&md5=a98f5f7160e7faa4ba14daf360062ea6

16. Badiru IO, Afolabi OE. Consumers’ Inclination to Utilize Online Marketing Portals for Agricultural Purchases in Lagos, Nigeria. Journal of Agricultural and Food Information [Internet]. 2016;17(4):290–9. Available from: https://www.scopus.com/inward/record.uri?eid=2-s2.0-84981719201&doi=10.1080%2f10496505.2016.1211015&partnerID=40&md5=fdf7a070240676463d661ab17c105860

17. Bahn RA, Abebe GK. Food retail expansion patterns in Sub-Saharan Africa and the Middle East and North Africa: Institutional and regional perspectives. Agribusiness. 2019;

18. Banerjee A, Hanna R, Kyle J, Olken BA, Sumarto S. Tangible Information and Citizen Empowerment: Identification Cards and Food Subsidy Programs in Indonesia. JOURNAL OF POLITICAL ECONOMY. 2018;126(2):451–91.

19. Banerji A, Meenakshi JV. Buyer collusion and efficiency of government intervention in wheat markets in northern India: An asymmetric structural auctions analysis. American Journal of Agricultural Economics [Internet]. 2004;86(1):236–53. Available from: https://www.scopus.com/inward/record.uri?eid=2-s2.0-1342289971&doi=10.1111%2fj.0092-5853.2004.00575.x&partnerID=40&md5=5f839b0df7da0dbc19cc19beda43ad0f

20. Barbier EB. Corruption, Poverty and Tropical Land Use. Journal of Sustainable Forestry [Internet]. 2012;31(4–5):319–39. Available from: https://www.scopus.com/inward/record.uri?eid=2-s2.0-84861940928&doi=10.1080%2f10549811.2011.588455&partnerID=40&md5=f00360cdb7e34076e2df8f843426284d

21. Barclay K, Cartwright I. Governance of tuna industries: The key to economic viability and sustainability in the Western and Central Pacific Ocean. MARINE POLICY. 2007;31(3):348–58.

22. Barnard C, O’Connor N. RUNNERS and RIDERS: The HORSEMEAT SCANDAL, EU LAW and MULTI-LEVEL ENFORCEMENT. Cambridge Law Journal [Internet]. 2017;76(1):116–44. Available from: https://www.scopus.com/inward/record.uri?eid=2-s2.0-85017179993&doi=10.1017%2fS000819731700006X&partnerID=40&md5=8fb00a6d955bbb88e2f2c35b29654829

23. Barnett J, Begen F, Howes S, Regan A, McConnon A, Marcu A, et al. Consumers’ confidence, reflections and response strategies following the horsemeat incident. Food Control [Internet]. 2016;59:721–30. Available from: https://www.scopus.com/inward/record.uri?eid=2-s2.0-84936133682&doi=10.1016%2fj.foodcont.2015.06.021&partnerID=40&md5=9452954a0c17e85030be19be3d3719a1

24. Baudoin MA, Sanchez AC, Fandohan B. Small scale farmers’ vulnerability to climatic changes in southern Benin: the importance of farmers’ perceptions of existing institutions. MITIGATION AND ADAPTATION STRATEGIES FOR GLOBAL CHANGE. 2014;19(8):1195–207.

25. Beekman G, Bulte EH, Nillesen EEM. Corruption and economic activity: Micro level evidence from rural Liberia. EUROPEAN JOURNAL OF POLITICAL ECONOMY. 2013;30:70–9.

26. Beg S. Tenancy and clientelism. Journal of Economic Behavior & Organization. 2021;186:201–26.

27. Bélair J. Farmland investments in Tanzania: The impact of protected domestic markets and patronage relations. World Development [Internet]. 2021;139. Available from: https://www.scopus.com/inward/record.uri?eid=2-s2.0-85097126273&doi=10.1016%2fj.worlddev.2020.105298&partnerID=40&md5=01e98056b293fa9aa5de176f3bd41a6e

28. Bellemare MF, Carnes N. Why do members of congress support agricultural protection? Food Policy [Internet]. 2015;50:20–34. Available from: https://www.scopus.com/inward/record.uri?eid=2-s2.0-84909619819&doi=10.1016%2fj.foodpol.2014.10.010&partnerID=40&md5=24c55587cab66688ee2c639409e60a28

29. Benbrook CM, Baker BP. Perspective on dietary risk assessment of pesticide residues in organic food. Sustainability (Switzerland) [Internet]. 2014;6(6):3552–70. Available from: https://www.scopus.com/inward/record.uri?eid=2-s2.0-84904299743&doi=10.3390%2fsu6063552&partnerID=40&md5=5cd3ac4e7acbe38aea1aacfb2b6f6dbe

30. Benjaminsen TA, Alinon K, Buhaug H, Buseth JT. Does climate change drive land-use conflicts in the sahel? Journal of Peace Research [Internet]. 2012;49(1):97–111. Available from: https://www.scopus.com/inward/record.uri?eid=2-s2.0-84856443719&doi=10.1177%2f0022343311427343&partnerID=40&md5=c411c77f0ee8ec85e93a122f2f023216

31. Benjaminsen TA, Maganga FP, Abdallah JM. The Kilosa Killings: Political Ecology of a Farmer-Herder Conflict in Tanzania. DEVELOPMENT AND CHANGE. 2009;40(3):423–45.

32. Bergeron H, Castel P, Saguy AC. A french paradox? Toward an Explanation of inconsistencies between Framing and Policies. French Politics, Culture and Society [Internet]. 2019;37(2):110–30. Available from: https://www.scopus.com/inward/record.uri?eid=2-s2.0-85077693808&doi=10.3167%2ffpcs.2019.370205&partnerID=40&md5=a277ad0178deb852cebc684e56906849

33. Beseng M. Cameroon’s choppy waters: The anatomy of fisheries crime in the maritime fisheries sector. Marine Policy [Internet]. 2019;108. Available from: https://www.scopus.com/inward/record.uri?eid=2-s2.0-85071402500&doi=10.1016%2fj.marpol.2019.103669&partnerID=40&md5=70194c1c7b40d6813e12c7146c9d5d75

34. Bhuyan S. Corporate political activities and oligopoly welfare loss. Review of Industrial Organization [Internet]. 2000;17(4):411–26. Available from: https://www.scopus.com/inward/record.uri?eid=2-s2.0-0033677250&doi=10.1023%2fA%3a1007843812053&partnerID=40&md5=d08003fb4713238c0fca2af4f4e062f1

35. Bimbo F, Bonanno A, Viscecchia R. An empirical framework to study food labelling fraud: an application to the Italian extra-virgin olive oil market. AUSTRALIAN JOURNAL OF AGRICULTURAL AND RESOURCE ECONOMICS. 2019;63(4):701–25.

36. Bødker M, Pisinger C, Toft U, Jørgensen T. The rise and fall of the world’s first fat tax. Health Policy. 2015;119(6):737–42.

37. Boerder K, Miller NA, Worm B. Global hot spots of transshipment of fish catch at sea. Science Advances [Internet]. 2018;4(7). Available from: https://www.scopus.com/inward/record.uri?eid=2-s2.0-85050812321&doi=10.1126%2fsciadv.aat7159&partnerID=40&md5=54ecbdfd9b5056acffba3701c466b43d

38. Bouzembrak Y, Marvin HJP. Prediction of food fraud type using data from Rapid Alert System for Food and Feed (RASFF) and Bayesian network modelling. Food Control [Internet]. 2016;61:180–7. Available from: https://www.scopus.com/inward/record.uri?eid=2-s2.0-84943559851&doi=10.1016%2fj.foodcont.2015.09.026&partnerID=40&md5=82a57397af175cb3ab4350b7e0518a8f

39. Brock C. Partisan polarization and corporate lobbying: information, demand, and conflict. Interest Groups and Advocacy [Internet]. 2021;10(2):95–113. Available from: https://www.scopus.com/inward/record.uri?eid=2-s2.0-85100825724&doi=10.1057%2fs41309-021-00112-5&partnerID=40&md5=74f29de0560e296cd182698dedd3a1a4

40. Bromley D, Foltz J. Sustainability under siege: Transport costs and corruption on West Africa’s trade corridors. Natural Resources Forum [Internet]. 2011;35(1):32–48. Available from: https://www.scopus.com/inward/record.uri?eid=2-s2.0-79951766643&doi=10.1111%2fj.1477-8947.2011.01342.x&partnerID=40&md5=544de2b8977c7571bbe07ec2ab5306eb

41. Brooks S, Elliott C, Spence M, Walsh C, Dean M. Four years post-horsegate: an update of measures and actions put in place following the horsemeat incident of 2013. NPJ SCIENCE OF FOOD. 2017;1(1).

42. Brown T. Legislative Capture: A Critical Consideration in the Commercial Determinants of Public Health. Journal of law and medicine [Internet]. 2019;26(4):764–85. Available from: http://www.ncbi.nlm.nih.gov/pubmed/31682356

43. Bulte EH, Damania R, López R. On the gains of committing to inefficiency: Corruption, deforestation and low land productivity in Latin America. Journal of Environmental Economics and Management [Internet]. 2007;54(3):277–95. Available from: https://www.scopus.com/inward/record.uri?eid=2-s2.0-35549011056&doi=10.1016%2fj.jeem.2007.05.002&partnerID=40&md5=7c76830d31c42bc602b72e76802c8d08

44. Bumblauskas D, Mann A, Dugan B, Rittmer J. A blockchain use case in food distribution: Do you know where your food has been? International Journal of Information Management [Internet]. 2020;52. Available from: https://www.scopus.com/inward/record.uri?eid=2-s2.0-85073210750&doi=10.1016%2fj.ijinfomgt.2019.09.004&partnerID=40&md5=c8a6bbb3ab5b3d6906056184bda70556

45. Burlandy L, Prado Alexandre-Weiss V, Silva Canella D, Feldenheimer Da Silva AC, Maranha Paes De Carvalho C, Rugani Ribeiro De Castro I. Obesity agenda in Brazil, conflicts of interest and corporate activity. Health Promotion International [Internet]. 2021;36(4):1186–97. Available from: https://www.scopus.com/inward/record.uri?eid=2-s2.0-85115005260&doi=10.1093%2fheapro%2fdaaa085&partnerID=40&md5=90cdce5cec08a883b7dfba80f98b21c5

46. Campbell I, Tranfaglia MA, Tham JC, Boese M. Precarious work and the reluctance to complain: Italian temporary migrant workers in Australia. Labour and Industry [Internet]. 2019 Jan 2;29(1):98–117. Available from: https://doi.org/10.1080/10301763.2018.1558895

47. Carey R, Caraher M, Lawrence M, Friel S. Opportunities and challenges in developing a whole-of-government national food and nutrition policy: Lessons from Australia’s National Food Plan. Public Health Nutrition [Internet]. 2016;19(1):3–14. Available from: https://www.scopus.com/inward/record.uri?eid=2-s2.0-84930986975&doi=10.1017%2fS1368980015001834&partnerID=40&md5=4c4845602329c471eef1ad32f608fbd3

48. Carrero GC, Fearnside PM, do Valle DR, de Souza Alves C. Deforestation Trajectories on a Development Frontier in the Brazilian Amazon: 35 Years of Settlement Colonization, Policy and Economic Shifts, and Land Accumulation. Environmental Management [Internet]. 2020;66(6):966–84. Available from: https://www.scopus.com/inward/record.uri?eid=2-s2.0-85091080983&doi=10.1007%2fs00267-020-01354-w&partnerID=40&md5=83793bd2c7901b5151b805c69968dd1a

49. Chang SC. The effects of trade liberalization on environmental degradation. Quality and Quantity [Internet]. 2015;49(1):235–53. Available from: https://www.scopus.com/inward/record.uri?eid=2-s2.0-84890462409&doi=10.1007%2fs11135-013-9984-4&partnerID=40&md5=91b0cb8c201e4875f02ad2dd68b986a3

50. Chapsos I, Hamilton S. Illegal fishing and fisheries crime as a transnational organized crime in Indonesia. TRENDS IN ORGANIZED CRIME. 2019;22(3):255–73.

51. Che FN, Strang KD, Vajjhala NR. Voice of farmers in the agriculture crisis in North-East Nigeria: Focus group insights from extension workers. International Journal of Development Issues [Internet]. 2020;19(1):43–61. Available from: https://www.scopus.com/inward/record.uri?eid=2-s2.0-85078895967&doi=10.1108%2fIJDI-08-2019-0136&partnerID=40&md5=277b8ea19a07230cf4448fdbbf23b776

52. Cheng H. Cheap capitalism: A sociological study of food crime in China. British Journal of Criminology. 2012;52(2):254–73.

53. Chilombo A. Multilevel governance of large-scale land acquisitions: a case study of the institutional politics of scale of the farm block program in Zambia. Land Use Policy [Internet]. 2021;107. Available from: https://www.scopus.com/inward/record.uri?eid=2-s2.0-85105320007&doi=10.1016%2fj.landusepol.2021.105518&partnerID=40&md5=ff9081570dc64483752c81925f5aab4b

54. Chirwa WC. Land use and extension services at wovwe rice scheme, Malawi. Development Southern Africa [Internet]. 2002;19(2):307–27. Available from: https://www.scopus.com/inward/record.uri?eid=2-s2.0-0036061958&doi=10.1080%2f03768350020013543&partnerID=40&md5=f2e1a2395617e6239ad9174862b3953a

55. Chukwuone NA, Agwu AE, Ozor N. Constraints and strategies toward effective cost-sharing of agricultural technology delivery in Nigeria. Journal of international agricultural and extension education [Internet]. 2006;13(1):29–42. Available from: http://ezproxy.library.yorku.ca/login?url=https://search.proquest.com/docview/36441582?accountid=15182

56. Cisneros-Montemayor AM, Cisneros-Mata MA, Harper S, Pauly D. Extent and implications of IUU catch in Mexico’s marine fisheries. MARINE POLICY. 2013;39:283–8.

57. Cissokho L, Haughton J, Makpayo K, Seck A. Why is agricultural trade within ECOWAS so high? Journal of African Economies [Internet]. 2013;22(1):22–51. Available from: https://www.scopus.com/inward/record.uri?eid=2-s2.0-84871596684&doi=10.1093%2fjae%2fejs015&partnerID=40&md5=875203fb72b4fb73963094cf3ae617bd

58. Collins R, Sun X. China’s grey channels as access points for foreign food products to the Chinese domestic market. China Information [Internet]. 2010;24(1):61–74. Available from: https://www.scopus.com/inward/record.uri?eid=2-s2.0-77749283153&doi=10.1177%2f0920203X09354962&partnerID=40&md5=4e1302c267323ad73243b452cb343b7d

59. Dandurand G. When Biopolitics Turn Digital: Transparency, Corruption, and Erasures from the Infrastructure of Rationing in Delhi. Political and Legal Anthropology Review [Internet]. 2019;42(2):268–82. Available from: http://ezproxy.library.yorku.ca/login?url=https://search.proquest.com/docview/2328359074?accountid=15182

60. Davies J, Ollus N. Labour exploitation as corporate crime and harm: outsourcing responsibility in food production and cleaning services supply chains. Crime, Law and Social Change [Internet]. 2019;72(1):87–106. Available from: https://www.scopus.com/inward/record.uri?eid=2-s2.0-85066156711&doi=10.1007%2fs10611-019-09841-w&partnerID=40&md5=91b575ba66484fe352c384ac5949e511

61. Davies J. Corporate harm and embedded labour exploitation in agri-food supply networks. European Journal of Criminology. 2020;17(1):70–85.

62. De Rosa M, Trabalzi F. Everybody does it, or how illegality is socially constructed in a southern Italian food network. JOURNAL OF RURAL STUDIES. 2016;45:303–11.

63. Deshingkar P, Johnson C, Farrington J. State transfers to the poor and back: The case of the food-for-work program in India. WORLD DEVELOPMENT. 2005;33(4):575–91.

64. Dodson EA, Fleming C, Boehmer TK, Haire-Joshu D, Luke DA, Brownson RC. Preventing childhood obesity through state policy: Qualitative assessment of enablers and barriers. Journal of Public Health Policy [Internet]. 2009;30(SUPPL. 1):S161–76. Available from: https://www.scopus.com/inward/record.uri?eid=2-s2.0-59849121321&doi=10.1057%2fjphp.2008.57&partnerID=40&md5=2430d1f7f9e68cfde7a852030fa1c0c9

65. Ebewore SO. Farmers’ perception of the state of infrastructure on agricultural production in Delta state, Nigeria. Rural Society [Internet]. 2021;30(1):32–44. Available from: https://www.scopus.com/inward/record.uri?eid=2-s2.0-85102686120&doi=10.1080%2f10371656.2021.1897239&partnerID=40&md5=f1a5761eabebd0397b17aa2cf24deb51

66. Ekbom A, Knutsson P, Ovuka M. Is sustainable development based on agriculture attainable in Kenya? A multidisciplinary case study of Muranga district. LAND DEGRADATION & DEVELOPMENT. 2001;12(5):435–47.

67. Elsen S, Fazzi L. Extending the concept of social farming: Rural development and the fight against organized crime in disadvantaged areas of southern Italy. Journal of Rural Studies [Internet]. 2021;84:100–7. Available from: https://www.scopus.com/inward/record.uri?eid=2-s2.0-85103644837&doi=10.1016%2fj.jrurstud.2021.03.009&partnerID=40&md5=9c46682513c622b3b85761a7fc7f40c1

68. Evans O, Alenoghena R. Corruption Effects on Nigeria: Aggregate and Sectoral Estimates Using VAR. Journal of Economic and Financial Studies [Internet]. 2015;3(3):41–8. Available from: https://search.ebscohost.com/login.aspx?direct=true&db=ecn&AN=1632667&site=ehost-live

69. Fazzi L, Elsen S. Actors in social agriculture cooperatives combating organized crime in southern italy: Cultivating the ground. Sustainability (Switzerland) [Internet]. 2020;12(21):1–11. Available from: https://www.scopus.com/inward/record.uri?eid=2-s2.0-85097776396&doi=10.3390%2fsu12219257&partnerID=40&md5=92b7b846f7a3662329c2d322db49b0e1

70. Fearnley L. Fake eggs: from counter-qualification to popular certification in China’s food safety crisis. BioSocieties [Internet]. 2021; Available from: https://www.scopus.com/inward/record.uri?eid=2-s2.0-85099372593&doi=10.1057%2fs41292-020-00211-7&partnerID=40&md5=41f4bc6d86238ced6fc518e042f11bbe

71. Fikselová M, Benešová L, Zajác P, Golian J, Čapla J. Food adulteration and safety regarding detected market cases and consumer opinions. Potravinarstvo Slovak Journal of Food Sciences [Internet]. 2020;14:417–28. Available from: https://www.scopus.com/inward/record.uri?eid=2-s2.0-85090277875&doi=10.5219%2f1345&partnerID=40&md5=7c4e2c9c9dc9605679bfa66b0de8cdd4

72. Fraser A. Ghosts in the vending machine: Expressing corporate power in Ireland’s food and drinks industry via the territorialization of selective openness. Human Geography(United Kingdom) [Internet]. 2021;14(1):110–22. Available from: https://www.scopus.com/inward/record.uri?eid=2-s2.0-85106404075&doi=10.1177%2f1942778620978212&partnerID=40&md5=7169514a888d1ccce8f7f2dad360a196

73. Friggeri FP. Primitive Accumulation, Mafia Capitalism, and the Campesino Population in Paraguay. Latin American Perspectives [Internet]. 2021;48(1):126–44. Available from: https://www.scopus.com/inward/record.uri?eid=2-s2.0-85096955961&doi=10.1177%2f0094582X20975002&partnerID=40&md5=38c9e74b2a1f2efd413ee9f8b72b2511

74. Gafițianu D, Borda D, Dumitrascu L, Adam R, Nicolau AI. A consumer’s perspective on the active role of online media endorsement of food integrity by connecting the actors involved. Annals of the University Dunarea de Jos of Galati, Fascicle VI: Food Technology [Internet]. 2020;44(2):137–58. Available from: https://www.scopus.com/inward/record.uri?eid=2-s2.0-85102726086&doi=10.35219%2fFOODTECHNOLOGY.2020.2.09&partnerID=40&md5=3e1c566f33c7b228fda7b28c340ca24b

75. Galinato GI, Galinato SP. The short-run and long-run effects of corruption control and political stability on forest cover. ECOLOGICAL ECONOMICS. 2013;89:153–61.

76. Gharib MH, Palm-Forster LH, Lybbert TJ, Messer KD. Fear of fraud and willingness to pay for hybrid maize seed in Kenya. Food Policy [Internet]. 2021;102. Available from: https://www.scopus.com/inward/record.uri?eid=2-s2.0-85103925385&doi=10.1016%2fj.foodpol.2021.102040&partnerID=40&md5=197f792c2748996a04bc9fe6461027e2

77. Gluszek S, Viollaz J, Mwinyihali R, Wieland M, Gore ML. Using conservation criminology to understand the role of restaurants in the urban wild meat trade. Conservation Science and Practice [Internet]. 2021 [cited 2021 Oct 19];3(5):e368. Available from: https://onlinelibrary.wiley.com/doi/abs/10.1111/csp2.368

78. Gomez M, Perdiguero J, Sanz A. Socioeconomic Factors Affecting Water Access in Rural Areas of Low and Middle Income Countries. WATER. 2019;11(2).

79. Goodall O. Rural criminal collaborations and the food crimes of the countryside: realist social relations theory of illicit venison production. Crime, Law and Social Change [Internet]. 2021; Available from: https://www.scopus.com/inward/record.uri?eid=2-s2.0-85112114291&doi=10.1007%2fs10611-021-09976-9&partnerID=40&md5=9271198d509db411254872cebb2626ad

80. Graham V, Sadie Y, Patel L. Social grants, food parcels and voting behaviour: a case study of three South African communities. TRANSFORMATION-CRITICAL PERSPECTIVES ON SOUTHERN AFRICA. 2016;91:106–35.

81. Grydehoj A, Nurdin N. Politics of technology in the informal governance of destructive fishing in Spermonde, Indonesia. GEOJOURNAL. 2016;81(2):281–92.

82. Guntzburger Y, Théolier J, Barrere V, Peignier I, Godefroy S, de Marcellis-Warin N. Food industry perceptions and actions towards food fraud: Insights from a pan-Canadian study. Food Control [Internet]. 2020;113. Available from: https://www.scopus.com/inward/record.uri?eid=2-s2.0-85081023666&doi=10.1016%2fj.foodcont.2020.107182&partnerID=40&md5=8fc1bab2895ebe064711556e700c6f0a

83. Gupta S. Worlds apart? Challenges of multi-agency partnership in participatory watershed development in Rajasthan, India. Development Studies Research [Internet]. 2014;1(1):100–12. Available from: https://www.scopus.com/inward/record.uri?eid=2-s2.0-85006410530&doi=10.1080%2f21665095.2014.938088&partnerID=40&md5=3b230284d69b8d4fc03716bd70e12ab1

84. Gylfason T. Resources, agriculture, and economic growth in economies in transition. KYKLOS. 2000;53(4):545–79.

85. Hanf M, Van-Melle A, Fraisse F, Roger A, Carme B, Nacher M. Corruption Kills: Estimating the Global Impact of Corruption on Children Deaths. PLOS ONE. 2011;6(11).

86. Han G, Yan S. Does Food Safety Risk Perception Affect the Public’s Trust in Their Government? An Empirical Study on a National Survey in China. INTERNATIONAL JOURNAL OF ENVIRONMENTAL RESEARCH AND PUBLIC HEALTH. 2019;16(11).

87. Hausermann H, Ferring D, Atosona B, Mentz G, Amankwah R, Chang A, et al. Land-grabbing, land-use transformation and social differentiation: Deconstructing ``small-scale{’’} in Ghana’s recent gold rush. WORLD DEVELOPMENT. 2018;108:103–14.

88. Hernandez-Aguado I, Chilet-Rosell E. Pathways of undue influence in health policy-making: a main actor’s perspective. JOURNAL OF EPIDEMIOLOGY AND COMMUNITY HEALTH. 2018;72(2):154–9.

89. Hobbs SH, Ricketts TC, Dodds JM, Milio N. Analysis of interest group influence on federal school meals regulations 1992 to 1996. Journal of Nutrition Education and Behavior [Internet]. 2004;36(2):90–8. Available from: https://www.scopus.com/inward/record.uri?eid=2-s2.0-1942535021&doi=10.1016%2fS1499-4046%2806%2960139-5&partnerID=40&md5=a2c1417fbeaaf03152893573849834d9

90. Hoff A, Lelieveldt H, van der Does R. A biased rapporteur or politics as usual? Reassessing the balance of interests in the EU food information labelling case. Journal of European Public Policy [Internet]. 2016;23(2):296–313. Available from: https://www.scopus.com/inward/record.uri?eid=2-s2.0-84955215768&doi=10.1080%2f13501763.2015.1051095&partnerID=40&md5=b7b8d8c41e90f8718134c803d6980363

91. Holden ST, Lunduka RW. Who benefit from Malawi’s targeted farm input subsidy program? Forum for development studies [Internet]. 2013;40(1):1–25. Available from: http://ezproxy.library.yorku.ca/login?url=https://search.proquest.com/docview/1316052319?accountid=15182

92. Hossain N, Kalita D. Moral economy in a global era: the politics of provisions during contemporary food price spikes. Journal of Peasant Studies [Internet]. 2014;41(5):815–31. Available from: https://www.scopus.com/inward/record.uri?eid=2-s2.0-84907576066&doi=10.1080%2f03066150.2014.895328&partnerID=40&md5=1f615622a02d98ed10431c9d4ea3813c

93. Hunt D. How food companies use social media to influence policy debates: A framework of Australian ultra-processed food industry Twitter data. Public Health Nutrition [Internet]. 2021;24(10):3124–35. Available from: https://www.scopus.com/inward/record.uri?eid=2-s2.0-85097259639&doi=10.1017%2fS1368980020003353&partnerID=40&md5=adae9f623965eb2b277afec27e2430ff

94. Hu Z, Zhang QF, Donaldson JA. Farmers’ cooperatives in china: A typology of fraud and failure. China Journal [Internet]. 2017;78. Available from: https://www.scopus.com/inward/record.uri?eid=2-s2.0-85032263418&doi=10.1086%2f691786&partnerID=40&md5=b9f43af487ced8df69af6d65e0e1158f

95. Ibrahim SS, Ibrahim A, Allah AN, Saulawa LA. Building of a community cattle ranch and radio frequency identification (RFID) technology as alternative methods of curtailing cattle rustling in Katsina State. PASTORALISM-RESEARCH POLICY AND PRACTICE. 2016;6.

96. Ingram V, Ewane M, Ndumbe LN, Awono A. Challenges to governing sustainable forest food: &ITIrvingia&IT spp. from southern Cameroon. FOREST POLICY AND ECONOMICS. 2017;84(SI):29–37.

97. Isaacs M, Witbooi E. Fisheries crime, human rights and small-scale fisheries in South Africa: A case of bigger fish to fry. Marine Policy [Internet]. 2019;105:158–68. Available from: https://www.scopus.com/inward/record.uri?eid=2-s2.0-85060328735&doi=10.1016%2fj.marpol.2018.12.023&partnerID=40&md5=f9905f8eb9e51a475f25333983630fb1

98. Ismaila S, Tanko M. Exploring relative deprivation theory in the rice industry: Planting for Food and Jobs (PFJ) in northern Ghana. Technology in Society [Internet]. 2021;65. Available from: https://www.scopus.com/inward/record.uri?eid=2-s2.0-85103953469&doi=10.1016%2fj.techsoc.2021.101556&partnerID=40&md5=c4fe92126d20c91af707a37f64800104

99. Jacoby HG, Mansuri G, Fatima F. Decentralizing corruption: Irrigation reform in Pakistan. Journal of Public Economics [Internet]. 2021;202. Available from: https://www.scopus.com/inward/record.uri?eid=2-s2.0-85115242790&doi=10.1016%2fj.jpubeco.2021.104499&partnerID=40&md5=dafce1beadd744377a52bb9f23fdbf05

100. Jaichuen N, Phulkerd S, Certthkrikul N, Sacks G, Tangcharoensathien V. Corporate political activity of major food companies in Thailand: An assessment and policy recommendations. Globalization and Health [Internet]. 2018;14(1). Available from: https://www.scopus.com/inward/record.uri?eid=2-s2.0-85057123906&doi=10.1186%2fs12992-018-0432-z&partnerID=40&md5=8bd33958ee095d807e1d91c8b909b4a1

101. Jeffrey C. Caste, class, and clientelism: A political economy of everyday corruption in rural north India. ECONOMIC GEOGRAPHY. 2002;78(1):21–41.

102. Kaitibie S, Munshi MH, Rakotoarisoa MA. Analysis of Food Imports in a Highly Import Dependent Economy. Review of Middle East Economics & Finance [Internet]. 2017;13(2):106–16. Available from: http://ezproxy.library.yorku.ca/login?url=https://search.proquest.com/docview/1945847651?accountid=15182

103. Kalaora L. Madness, corruption and exile: On Zimbabwe’s remaining white commercial farmers. Journal of Southern African Studies [Internet]. 2011;37(4):747–62. Available from: https://www.scopus.com/inward/record.uri?eid=2-s2.0-84858374949&doi=10.1080%2f03057070.2011.609341&partnerID=40&md5=f5ad4df30a9edf97381ddd68dc767866

104. Kansanga MM, Luginaah I. Agrarian livelihoods under siege: Carbon forestry, tenure constraints and the rise of capitalist forest enclosures in Ghana. WORLD DEVELOPMENT. 2019;113:131–42.

105. Kantel AJ. Fishing for Power: Incursions of the Ugandan Authoritarian State. Annals of the American Association of Geographers [Internet]. 2019;109(2):443–55. Available from: https://www.scopus.com/inward/record.uri?eid=2-s2.0-85060933856&doi=10.1080%2f24694452.2018.1527679&partnerID=40&md5=0da5ca6abe14bb0bae4e0a4726267141

106. Kassem H, Hussein M, Ismail H. Toward Fraudulent Pesticides in Rural Areas: Do Farmers’ Recognition and Purchasing Behaviors Matter? AGRONOMY-BASEL. 2021;11(9).

107. Kaul T. Household responses to food subsidies: Evidence from India. Economic Development and Cultural Change. 2018;67(1):95–129.

108. Kendall H, Kuznesof S, Dean M, Chan MY, Clark B, Home R, et al. Chinese consumer’s attitudes, perceptions and behavioural responses towards food fraud. Food Control [Internet]. 2019;95:339–51. Available from: https://www.scopus.com/inward/record.uri?eid=2-s2.0-85053210940&doi=10.1016%2fj.foodcont.2018.08.006&partnerID=40&md5=b9eb8bb10ec15e0f04a6e12603be91f6

109. Kendall H, Naughton P, Kuznesof S, Raley M, Dean M, Clark B, et al. Food fraud and the perceived integrity of European food imports into China. PLoS ONE [Internet]. 2018;13(5). Available from: https://www.scopus.com/inward/record.uri?eid=2-s2.0-85047484951&doi=10.1371%2fjournal.pone.0195817&partnerID=40&md5=3f2741895249543e1333049612f33cb2

110. Kimanthi H, Hebinck P. ‘Castle in the sky’: The anomaly of the millennium villages project fixing food and markets in Sauri, western Kenya. Journal of Rural Studies. 2018;57:157–70.

111. Kopytko N. Change and transition: the climate of Ukraine’s agri-food sector. Climate Policy. 2016;16(1):68–87.

112. Koubová J, Samková E, Hasonová L. Food fraud detection by Czech Agricultural and Food Inspection Authority in retail market. British Food Journal [Internet]. 2018;120(4):930–8. Available from: https://www.scopus.com/inward/record.uri?eid=2-s2.0-85045020078&doi=10.1108%2fBFJ-07-2017-0367&partnerID=40&md5=eb7668503b1dec67fb5fd8bb9d322a14

113. Kowalska A. The study of the intersection between food FR aud / adulter ation and authenticity. Acta Universitatis Agriculturae et Silviculturae Mendelianae Brunensis [Internet]. 2018;66(5):1275–86. Available from: https://www.scopus.com/inward/record.uri?eid=2-s2.0-85056270819&doi=10.11118%2factaun201866051275&partnerID=40&md5=a929a1d50ade81c21c9640e8e7d16ff7

114. Kroetz K, Luque GM, Gephart JA, Jardine SL, Lee P, Moore KC, et al. Consequences of seafood mislabeling for marine populations and fisheries management. Proceedings of the National Academy of Sciences of the United States of America [Internet]. 2020;117(48):30318–23. Available from: https://www.scopus.com/inward/record.uri?eid=2-s2.0-85097211489&doi=10.1073%2fpnas.2003741117&partnerID=40&md5=3b022ca48abab4958413d9b5dbe2028e

115. Kumar B, Mohanty B. Public distribution system in rural India: Implications for food safety and consumer protection. In 2012. p. 232–8.

116. Lammer C. Distancing The Regulating State: Corruption, Transparency, And rhe Puzzle of Personal Relatedness In A Food Network In Sichuan. Urban Anthropology and Studies of Cultural Systems and World Economic Development [Internet]. 2017;47(3/4):369. Available from: http://ezproxy.library.yorku.ca/login?url=https://search.proquest.com/docview/2230824658?accountid=15182

117. Lander CD. Adaptive strategies of smaller foreign investors in the Russian agricultural sector: identity, narrative and performance. JOURNAL OF PEASANT STUDIES. 2019;46(1):165–87.

118. Larmour P. From clean up to FICAC: Anti-corruption in Fiji’s post coup politics. CRIME LAW AND SOCIAL CHANGE. 2010;53(1, SI):55–66.

119. Lauber K, Rutter H, Gilmore AB. Big food and the World Health Organization: a qualitative study of industry attempts to influence global-level non-communicable disease policy. BMJ Glob Health [Internet]. 2021 Jun [cited 2021 Sep 7];6(6):e005216. Available from: https://gh.bmj.com/lookup/doi/10.1136/bmjgh-2021-005216

120. Lee B, Fenoff R, Spink J. Routine activities theory and food fraud victimization. Security Journal [Internet]. 2021; Available from: https://www.scopus.com/inward/record.uri?eid=2-s2.0-85102363303&doi=10.1057%2fs41284-021-00287-1&partnerID=40&md5=0abc2e2c009bb99bf263db8dcc38d82d

121. Levy I, Kerschke-Risch P. Attitudes toward food fraud in Israel and Germany. British Food Journal [Internet]. 2020;122(7):2219–32. Available from: https://www.scopus.com/inward/record.uri?eid=2-s2.0-85083576528&doi=10.1108%2fBFJ-10-2019-0785&partnerID=40&md5=29294dfe185979b0fa9e35b9db0829c3

122. Lio MC, Hu JL. Governance and agricultural production efficiency: A cross-country aggregate frontier analysis. Journal of Agricultural Economics [Internet]. 2009;60(1):40–61. Available from: https://www.scopus.com/inward/record.uri?eid=2-s2.0-58149513397&doi=10.1111%2fj.1477-9552.2008.00172.x&partnerID=40&md5=a11edf56862a234f6a9db14e084551fd

123. Liu CY. Institutional Isomorphism and Food Fraud: A Longitudinal Study of the Mislabeling of Rice in Taiwan. Journal of Agricultural and Environmental Ethics. 2016;29(4):607–30.

124. Liu Y, Liu F, Zhang J, Gao J. Insights into the nature of food safety issues in Beijing through content analysis of an Internet database of food safety incidents inChina. Food Control [Internet]. 2015;51:206–11. Available from: https://www.scopus.com/inward/record.uri?eid=2-s2.0-84949116415&doi=10.1016%2fj.foodcont.2014.11.017&partnerID=40&md5=f31b238bcb1aed1b38d948ea6df7faeb

125. Lord N, Elizondo CJF, Spencer J. The dynamics of food fraud: The interactions between criminal opportunity and market (dys)functionality in legitimate business. CRIMINOLOGY & CRIMINAL JUSTICE. 2017;17(5):605–23.

126. Lovett N, Xue Y. Have electronic benefits cards improved food access for food stamp recipients? JOURNAL OF ECONOMIC STUDIES. 2017;44(6):958–75.

127. Mangla SK, Kazancoglu Y, Ekinci E, Liu M, Özbiltekin M, Sezer MD. Using system dynamics to analyze the societal impacts of blockchain technology in milk supply chainsrefer. Transportation Research Part E: Logistics and Transportation Review [Internet]. 2021;149. Available from: https://www.scopus.com/inward/record.uri?eid=2-s2.0-85103011950&doi=10.1016%2fj.tre.2021.102289&partnerID=40&md5=a7527014d3671d34785d03867d06a049

128. Mariath AB, Martins APB. Sugary drinks taxation: industry’s lobbying strategies, practices and arguments in the Brazilian Legislature. Public Health Nutr. 2021;1–10.

129. Martinez JC. Site of Resistance or Apparatus of Acquiescence? Tactics at the Bakery. MIDDLE EAST LAW AND GOVERNANCE. 2018;10(2):160–84.

130. Marvin HJP, Bouzembrak Y, Janssen EM, van der Fels- Klerx HJ, van Asselt ED, Kleter GA. A holistic approach to food safety risks: Food fraud as an example. Food Research International [Internet]. 2016;89:463–70. Available from: https://www.scopus.com/inward/record.uri?eid=2-s2.0-84995520513&doi=10.1016%2fj.foodres.2016.08.028&partnerID=40&md5=ec58d79055544468bcc50907a5fc1415

131. McElwee G, Smith R, Lever J. Illegal activity in the UK halal (sheep) supply chain: Towards greater understanding. Food Policy [Internet]. 2017;69:166–75. Available from: https://www.scopus.com/inward/record.uri?eid=2-s2.0-85017267556&doi=10.1016%2fj.foodpol.2017.04.006&partnerID=40&md5=eb2a6b3636c674cfdc173d286e8c8821

132. Meenakshi JV, Banerji A. The unsupportable support price: An analysis of collusion and government intervention in paddy auction markets in North India. Journal of Development Economics [Internet]. 2005;76(2):377–403. Available from: https://www.scopus.com/inward/record.uri?eid=2-s2.0-10944260130&doi=10.1016%2fj.jdeveco.2004.02.001&partnerID=40&md5=8c3edc1083bada1de63d968cd8652c49

133. Mehta A, Jha S. Corruption, food subsidies, and opacity: Evidence from the Philippines. Economics Letters. 2012;117(3):708–11.

134. Mehta A, Jha S. Pilferage from opaque food subsidy programs: Theory and evidence. Food Policy. 2014;45:69–79.

135. Menon S. Aadhaar-based Biometric Authentication for PDS and Food Security: Observations on Implementation in Jharkhand’s Ranchi District. Indian Journal of Human Development [Internet]. 2017;11(3):387–401. Available from: https://www.scopus.com/inward/record.uri?eid=2-s2.0-85063625445&doi=10.1177%2f0973703017748384&partnerID=40&md5=13672b967910febeffb517970106b9aa

136. Mensah N, Amrago E, Asare J, Tutu F, Donkor A. Poultry farmers willingness to pay for agricultural tax: evidence from the Bono region, Ghana. WORLD JOURNAL OF ENTREPRENEURSHIP MANAGEMENT AND SUSTAINABLE DEVELOPMENT. 2021;17(2):290–306.

137. Mialon M, Charry DAG, Cediel G, Crosbie E, Scagliusi FB, Tamayo EMP. ‘I had never seen so many lobbyists’: Food industry political practices during the development of a new nutrition front-of-pack labelling system in Colombia. Public Health Nutrition [Internet]. 2021;24(9):2737–45. Available from: https://www.scopus.com/inward/record.uri?eid=2-s2.0-85092472140&doi=10.1017%2fS1368980020002268&partnerID=40&md5=6b5fa7a17a2c34a3b65660bbcd1b7d7e

138. Mialon M, Gaitan Charry DA, Cediel G, Crosbie E, Baeza Scagliusi F, Pérez Tamayo EM. ‘the architecture of the state was transformed in favour of the interests of companies’: Corporate political activity of the food industry in Colombia. Globalization and Health [Internet]. 2020;16(1). Available from: https://www.scopus.com/inward/record.uri?eid=2-s2.0-85092534307&doi=10.1186%2fs12992-020-00631-x&partnerID=40&md5=0161a4a45e1362c2dc0acdc5c013a83b

139. Mialon M, Mialon J. Analysis of corporate political activity strategies of the food industry: Evidence from France. Public Health Nutrition [Internet]. 2018;21(18):3407–21. Available from: https://www.scopus.com/inward/record.uri?eid=2-s2.0-85049866651&doi=10.1017%2fS1368980018001763&partnerID=40&md5=17c50f5d60b802efaa7df828b8a2433a

140. Mialon M, Mialon J. Corporate political activity of the dairy industry in France: An analysis of publicly available information. Public Health Nutrition [Internet]. 2017;20(13):2432–9. Available from: https://www.scopus.com/inward/record.uri?eid=2-s2.0-85022051920&doi=10.1017%2fS1368980017001197&partnerID=40&md5=60d0cb4f62d204c4988f951c9ff1dd6f

141. Mialon M, Swinburn B, Allender S, Sacks G. ‘Maximising shareholder value’: a detailed insight into the corporate political activity of the Australian food industry. Australian and New Zealand Journal of Public Health [Internet]. 2017;41(2):165–71. Available from: https://www.scopus.com/inward/record.uri?eid=2-s2.0-85010223807&doi=10.1111%2f1753-6405.12639&partnerID=40&md5=2a51505e6a319a1be91d0416873fa34a

142. Mialon M, Swinburn B, Wate J, Tukana I, Sacks G. Analysis of the corporate political activity of major food industry actors in Fiji. Globalization and Health [Internet]. 2016;12(1). Available from: https://www.scopus.com/inward/record.uri?eid=2-s2.0-84968764845&doi=10.1186%2fs12992-016-0158-8&partnerID=40&md5=f76403a26481e7cd7c07e7491643b25a

143. Montgomery R, Sumarto S, Mawardi S, Usman S, Toyamah N, Febriany V, et al. Deregulation of Indonesia’s interregional agricultural trade. Bulletin of Indonesian Economic Studies [Internet]. 2002;38(1):93–117. Available from: https://www.scopus.com/inward/record.uri?eid=2-s2.0-0036224951&doi=10.1080%2f000749102753620301&partnerID=40&md5=6c36ee1e4094989a5bdecd095a2d0b78

144. Moreira MJ, García-Díez J, de Almeida JMMM, Saraiva C. Consumer knowledge about food labeling and fraud. Foods [Internet]. 2021;10(5). Available from: https://www.scopus.com/inward/record.uri?eid=2-s2.0-85106975038&doi=10.3390%2ffoods10051095&partnerID=40&md5=d1a400cb1b76c378ad17c10855db399c

145. Mulligan C, Jawad A, Kent MP, Vanderlee L, L’Abbé MR. Stakeholder interactions with the federal government related to Bill S-228 and marketing to kids in Canada: a quantitative descriptive study. CMAJ open [Internet]. 2021;9(1):E280–7. Available from: https://www.scopus.com/inward/record.uri?eid=2-s2.0-85107911926&doi=10.9778%2fcmajo.20200086&partnerID=40&md5=28593f9ff69ab385154ed98aa5cead7c

146. Nading A. Orientation and Crafted Bureaucracy: Finding Dignity in Nicaraguan Food Safety. AMERICAN ANTHROPOLOGIST. 2017;119(3):478–90.

147. Nagavarapu S, Sekhri S. Informal monitoring and enforcement mechanisms in public service delivery: Evidence from the public distribution system in India. Journal of Development Economics. 2016;121:63–78.

148. Naqvi N. Finance and Industrial Policy in Unsuccessful Developmental States: The Case of Pakistan. DEVELOPMENT AND CHANGE. 2018;49(4):1064–92.

149. Neumann K, Stehfest E, Verburg PH, Siebert S, Muller C, Veldkamp T. Exploring global irrigation patterns: A multilevel modelling approach. AGRICULTURAL SYSTEMS. 2011;104(9):703–13.

150. Ng S, Kelly B, Yeatman H, Swinburn B, Karupaiah T. Tracking progress from policy development to implementation: A case study on adoption of mandatory regulation for nutrition labelling in malaysia. Nutrients [Internet]. 2021;13(2):1–18. Available from: https://www.scopus.com/inward/record.uri?eid=2-s2.0-85099972534&doi=10.3390%2fnu13020457&partnerID=40&md5=881d89599e3e8f093025e00382ca655f

151. Ogunniyi AI, Mavrotas G, Olagunju KO, Fadare O, Adedoyin R. Governance quality, remittances and their implications for food and nutrition security in Sub-Saharan Africa. WORLD DEVELOPMENT. 2020;127.

152. Oliva M. Money laundering, food activities and mafia: evidences from the Italian provinces. Journal of Money Laundering Control [Internet]. 2021; Available from: https://www.scopus.com/inward/record.uri?eid=2-s2.0-85115057111&doi=10.1108%2fJMLC-08-2021-0085&partnerID=40&md5=14348edd52d9b3333798b663d67b0398

153. öNDER H. The impact of corruption on food security from a macro perspective. Future of Food: Journal on Food, Agriculture and Society [Internet]. 2021;9(1):1–11. Available from: https://www.scopus.com/inward/record.uri?eid=2-s2.0-85104740851&doi=10.17170%2fkobra-202011192215&partnerID=40&md5=22baee488b454b44486143b854a19283

154. Osabohien R, Osabuohien E, Ohalete P. Agricultural sector performance, institutional framework and food security in Nigeria. Bio-based and Applied Economics [Internet]. 2019;8(2):161–78. Available from: https://www.scopus.com/inward/record.uri?eid=2-s2.0-85085337459&doi=10.13128%2fbae-8929&partnerID=40&md5=a0f4eea31c345744d13cd46013099941

155. Pailler S. Re-election incentives and deforestation cycles in the Brazilian Amazon. Journal of Environmental Economics and Management [Internet]. 2018;88:345–65. Available from: https://www.scopus.com/inward/record.uri?eid=2-s2.0-85041410667&doi=10.1016%2fj.jeem.2018.01.008&partnerID=40&md5=f7747151bdaf1a44bb9dae87c2186fd9

156. Panigrati S, Pathak N. Securing Food Through the Public Distribution System: Evidence from Odisha. GLOBAL SOCIAL WELFARE. 2018;5(2):117–23.

157. Parkinson S. Modes of influence: Participation in publicly administered development programmes. Public Administration and Development [Internet]. 2009;29(2):145–54. Available from: https://www.scopus.com/inward/record.uri?eid=2-s2.0-69949111676&doi=10.1002%2fpad.525&partnerID=40&md5=ceed05e9694f1cfb9b782411498848f6

158. Pavez I, Codron JM, Lubello P, Florêncio MC. Biosecurity institutions and the choice of contracts in international fruit supply chains. Agricultural Systems [Internet]. 2019;176. Available from: https://www.scopus.com/inward/record.uri?eid=2-s2.0-85071883441&doi=10.1016%2fj.agsy.2019.102668&partnerID=40&md5=62cf80696ccbaed1f38c1a290732efeb

159. Perone G. The impact of agribusiness crimes on food prices: evidence from Italy. ECONOMIA POLITICA [Internet]. 2020;(37):877–909. Available from: https://link.springer.com/article/10.1007/s40888-019-00165-5

160. Pittman J, Wabnitz CCC, Blasiak R. A global assessment of structural change in development funding for fisheries. MARINE POLICY. 2019;109.

161. Prishchepov AV, Ponkina EV, Sun Z, Bavorova M, Yekimovskaja OA. Revealing the intentions of farmers to recultivate abandoned farmland: A case study of the Buryat Republic in Russia. Land Use Policy [Internet]. 2021;107. Available from: https://www.scopus.com/inward/record.uri?eid=2-s2.0-85107639626&doi=10.1016%2fj.landusepol.2021.105513&partnerID=40&md5=2ed1c3f3267f923e3b2a361c738a1e86

162. Quandt SA, Arnold TJ, Mora DC, Sandberg JC, Daniel SS, Arcury TA. Hired Latinx child farm labor in North Carolina: The demand-support-control model applied to a vulnerable worker population. AMERICAN JOURNAL OF INDUSTRIAL MEDICINE. 2019;62(12):1079–90.

163. Raifu IA, Aminu A. Financial development and agricultural performance in Nigeria: what role do institutions play? Agricultural Finance Review [Internet]. 2020;80(2):231–54. Available from: https://www.scopus.com/inward/record.uri?eid=2-s2.0-85076530158&doi=10.1108%2fAFR-06-2018-0045&partnerID=40&md5=6d5db46f05b42300f97f7398fccb00ad

164. Rezazade F, Summers J, Lai Teik DO. A holistic approach to food fraud vulnerability assessment. Food Control [Internet]. 2021;131. Available from: https://www.scopus.com/inward/record.uri?eid=2-s2.0-85111113596&doi=10.1016%2fj.foodcont.2021.108440&partnerID=40&md5=48769d808f8e7180cca150ab19e0023f

165. Riley L, Chilanga E. `Things are not working now’: poverty, food insecurity and perceptions of corruption in urban Malawi. JOURNAL OF CONTEMPORARY AFRICAN STUDIES. 2018;36(4, SI):484–98.

166. Ringsberg HA. Implementation of global traceability standards: incentives and opportunities. BRITISH FOOD JOURNAL. 2015;117(7):1826–42.

167. Ritten C, Thunstrom L, Ehmke M, Beiermann J, McLeod D. International honey laundering and consumer willingness to pay a premium for local honey: an experimental study. AUSTRALIAN JOURNAL OF AGRICULTURAL AND RESOURCE ECONOMICS. 2019;63(4):726–41.

168. Rizzuti A. Organised food crime: an analysis of the involvements of organised crime groups in the food sector in England and Italy. Crime, Law and Social Change [Internet]. 2021; Available from: https://www.scopus.com/inward/record.uri?eid=2-s2.0-85110600716&doi=10.1007%2fs10611-021-09975-w&partnerID=40&md5=536e9ba8138fce7a8638368642c0ea0d

169. Robertson NM, Sacks G, Miller PG. The revolving door between government and the alcohol, food and gambling industries in Australia. Public Health Research and Practice [Internet]. 2019;29(3). Available from: https://www.scopus.com/inward/record.uri?eid=2-s2.0-85072774297&doi=10.17061%2fphrp2931921&partnerID=40&md5=4f49b1c0e2f3a0647a28cc2aad53567a

170. Robson K, Dean M, Brooks S, Haughey S, Elliott C. A 20-year analysis of reported food fraud in the global beef supply chain. Food Control [Internet]. 2020;116. Available from: https://www.scopus.com/inward/record.uri?eid=2-s2.0-85084356592&doi=10.1016%2fj.foodcont.2020.107310&partnerID=40&md5=a1150fff8c25786fd298dab35764eddc

171. Rocchi B, Romano D, Sadiddin A, Stefani G. Assessing the economy-wide impact of food fraud: A SAM-based counterfactual approach. Agribusiness [Internet]. 2020;36(2):167–91. Available from: https://www.scopus.com/inward/record.uri?eid=2-s2.0-85077846931&doi=10.1002%2fagr.21633&partnerID=40&md5=cc8ff4c1bc479da624dfa3f22043e88a

172. Ruslan AAA, Kamarulzaman NH, Sanny M. Muslim consumers’ awareness and perception of Halal food fraud. International Food Research Journal [Internet]. 2018;25:S87–96. Available from: https://www.scopus.com/inward/record.uri?eid=2-s2.0-85067253987&partnerID=40&md5=8f29c0243978555947cf3f0d7e053ae9

173. Sam K, Coulon F, Prpich G. Use of stakeholder engagement to support policy transfer: A case of contaminated land management in Nigeria. Environmental Development. 2017;24:50–62.

174. Schaefer KA, Scheitrum D, Nes K. International sourcing decisions in the wake of a food scandal. Food Policy [Internet]. 2018;81:48–57. Available from: https://www.scopus.com/inward/record.uri?eid=2-s2.0-85054435511&doi=10.1016%2fj.foodpol.2018.10.002&partnerID=40&md5=04e7b46271d851302ac4c20b406dbae3

175. Sebhatu KT, Gezahegn TW, Berhanu T, Maertens M, Van Passel S, D’Haese M. Conflict, fraud, and distrust in Ethiopian agricultural cooperatives. Journal of Co-operative Organization and Management [Internet]. 2020;8(1). Available from: https://www.scopus.com/inward/record.uri?eid=2-s2.0-85083775610&doi=10.1016%2fj.jcom.2020.100106&partnerID=40&md5=77557a7f67b12cbaa0bd1018414337ab

176. Shareef MA, Dwivedi YK, Kumar V, Mahmud R, Hughes DL, Rana NP, et al. The inherent tensions within sustainable supply chains: a case study from Bangladesh. PRODUCTION PLANNING & CONTROL.

177. Sheingate A, Scatterday A, Martin B, Nachman K. Post-exceptionalism and corporate interests in US agricultural policy. Journal of European Public Policy [Internet]. 2017;24(11):1641–57. Available from: https://www.scopus.com/inward/record.uri?eid=2-s2.0-85020735914&doi=10.1080%2f13501763.2017.1334082&partnerID=40&md5=8e897464b92fb5a5e921536d389159df

178. Shinn GC, Richard K, Rahmat F, Gary A, Briers E. Understanding afghan opinion leaders’ viewpoints about post-conflict foreign agricultural development: a case study in Herat Province, Afghanistan. Journal of international agricultural and extension education [Internet]. 2012;19(2). Available from: http://ezproxy.library.yorku.ca/login?url=https://search.proquest.com/docview/1124749266?accountid=15182

179. Shonhe T, Scoones I, Murimbarimba F. Medium-scale commercial agriculture in Zimbabwe: The experience of A2 resettlement farms. Journal of Modern African Studies [Internet]. 2020;58(4):601–26. Available from: https://www.scopus.com/inward/record.uri?eid=2-s2.0-85100597051&doi=10.1017%2fS0022278X20000385&partnerID=40&md5=fe2c27ab4bac7d1b1282363f91254647

180. Silvis ICJ, van Ruth SM, van der Fels-Klerx HJ, Luning PA. Assessment of food fraud vulnerability in the spices chain: An explorative study. Food Control [Internet]. 2017;81:80–7. Available from: https://www.scopus.com/inward/record.uri?eid=2-s2.0-85019999880&doi=10.1016%2fj.foodcont.2017.05.019&partnerID=40&md5=a3fd181ab5e94557d18a9602178bbfbf

181. Singh DR, Sunuwar DR, Shah SK, Sah LK, Karki K, Sah RK. Food insecurity during COVID-19 pandemic: A genuine concern for people from disadvantaged community and low-income families in Province 2 of Nepal. PLoS ONE [Internet]. 2021;16(July). Available from: https://www.scopus.com/inward/record.uri?eid=2-s2.0-85110959477&doi=10.1371%2fjournal.pone.0254954&partnerID=40&md5=ff6131590e853e568335ba79b9392351

182. Slangen LHG, Suchanek P, van Kooten GC. Trust in countries in transition: Empirical evidence from agriculture. International Journal of Social Economics [Internet]. 2003;30(9/10):1095–109. Available from: http://ezproxy.library.yorku.ca/login?url=https://search.proquest.com/docview/274622579?accountid=15182

183. Smith LED, Khushk AM, Stockbridge M. Case studies of corruption in agricultural markets in sindh province, pakistan, and implications for market liberalization. Journal of International Food and Agribusiness Marketing [Internet]. 2000;11(1):19–42. Available from: https://www.scopus.com/inward/record.uri?eid=2-s2.0-0033275558&doi=10.1300%2fJ047v11n01_02&partnerID=40&md5=8ffb01e52f0254f7790fc1cfaea34a76

184. Smith R. Documenting entrepreneurial opportunism in action A case study of sheep theft in the UK from a food supply chain perspective. BRITISH FOOD JOURNAL. 2017;119(1):105–21.

185. Soon JM, Krzyzaniak SC, Shuttlewood Z, Smith M, Jack L. Food fraud vulnerability assessment tools used in food industry. Food Control [Internet]. 2019;101:225–32. Available from: https://www.scopus.com/inward/record.uri?eid=2-s2.0-85063331357&doi=10.1016%2fj.foodcont.2019.03.002&partnerID=40&md5=05925becefbc416167d1c8b5f7227e62

186. Soyer B, Leloudas G, Miller D. Tackling IUU Fishing: Developing a Holistic Legal Response. TRANSNATIONAL ENVIRONMENTAL LAW. 2018;7(1):139–63.

187. Spink J, Moyer DC, Speier-Pero C. Introducing the Food Fraud Initial Screening model (FFIS). FOOD CONTROL. 2016;69:306–14.

188. Spink J, Embarek PB, Savelli CJ, Bradshaw A. Global perspectives on food fraud: results from a WHO survey of members of the International Food Safety Authorities Network (INFOSAN). npj Science of Food [Internet]. 2019;3(1). Available from: https://www.scopus.com/inward/record.uri?eid=2-s2.0-85073535442&doi=10.1038%2fs41538-019-0044-x&partnerID=40&md5=f0f1ee0af6bf6f3ce6d49d142d08be0f

189. Spink J, Bedard B, Keogh J, Moyer DC, Scimeca J, Vasan A. International Survey of Food Fraud and Related Terminology: Preliminary Results and Discussion. JOURNAL OF FOOD SCIENCE. 2019;84(10):2705–18.

190. Stamatis C, Sarri CA, Moutou KA, Argyrakoulis N, Galara I, Godosopoulos V, et al. What do we think we eat? Single tracing method across foodstuff of animal origin found in Greek market. Food Research International [Internet]. 2015;69:151–5. Available from: https://www.scopus.com/inward/record.uri?eid=2-s2.0-84920901683&doi=10.1016%2fj.foodres.2014.12.033&partnerID=40&md5=2b8cf3a41b9b0e01aa8c771ede6ce232

191. Standing A. MIRAGE OF PIRATES: STATE-CORPORATE CRIME IN WEST AFRICA’S FISHERIES. STATE CRIME. 2015;4(2):175–97.

192. Stanikzai AN, Ali F, Kamarulzaman NH. Vulnerabilities of wheat crop farmers in war zone. Food Research [Internet]. 2021;5(2):427–39. Available from: https://www.scopus.com/inward/record.uri?eid=2-s2.0-85105679807&doi=10.26656%2ffr.2017.5%282%29.506&partnerID=40&md5=2902436993652e29ff317ac74a2fd5ff

193. Sugden G. Farm Crime: Out of Sight, Out of Mind: A Study of Crime on Farms in the County of Rutland, England. Crime Prevention and Community Safety. 1999;1(3):29–36.

194. Sulle E. Social differentiation and the politics of land: Sugar cane outgrowing in Kilombero, Tanzania. Journal of Southern African Studies [Internet]. 2017;43(3):517–33. Available from: https://www.scopus.com/inward/record.uri?eid=2-s2.0-84987858721&doi=10.1080%2f03057070.2016.1215171&partnerID=40&md5=3b78a98544161fc3fa1fad500723650f

195. Sullivan L. Identity, Territory and Land Conflict in Brazil. Development and Change [Internet]. 2013;44(2):451–71. Available from: https://www.scopus.com/inward/record.uri?eid=2-s2.0-84875080743&doi=10.1111%2fdech.12010&partnerID=40&md5=ee6508178cb605c6a0f754d0c49bf1fc

196. Sundstrom A. Covenants with broken swords: Corruption and law enforcement in governance of the commons. GLOBAL ENVIRONMENTAL CHANGE-HUMAN AND POLICY DIMENSIONS. 2015;31:253–62.

197. Sunge R, Ngepah N. Agricultural trade liberalization, regional trade agreements and agricultural technical efficiency in Africa. OUTLOOK ON AGRICULTURE. 2019;

198. Tagliarino NK, Bununu YA, Micheal MO, De Maria M, Olusanmi A. Compensation for Expropriated Community Farmland in Nigeria: An In-Depth Analysis of the Laws and Practices Related to Land Expropriation for the Lekki Free Trade Zone in Lagos. LAND. 2018;7(1).

199. Tahkapaa S, Maijala R, Korkeala H, Nevas M. Patterns of food frauds and adulterations reported in the EU rapid alert system for food and feed and in Finland. FOOD CONTROL. 2015;47:175–84.

200. Thomas J, Thomas LT. Computer fraud perpetrated against small independent food retailers during the direct store delivery process. Journal of Small Business Management [Internet]. 1992;30(4):54. Available from: http://ezproxy.library.yorku.ca/login?url=https://search.proquest.com/docview/220954564?accountid=15182

201. Tibugari H, Chikasha T, Manyeruke N, Mathema N, Musara JP, Dlamini D, et al. Poor maize productivity in Zimbabwe: Can collusion in pricing by seed houses be the cause? Cogent Food and Agriculture [Internet]. 2019;5(1). Available from: https://www.scopus.com/inward/record.uri?eid=2-s2.0-85092662584&doi=10.1080%2f23311932.2019.1682230&partnerID=40&md5=6dd4b2018e7703d78cd5ac11840db112

202. Tracy M. Multimodality, Transparency, and Food Safety in China. POLAR-POLITICAL AND LEGAL ANTHROPOLOGY REVIEW. 2016;39(1):34–53.

203. Traikova D, Moellers J, Petrick M. Go west? Emigration intentions of young Bulgarian agricultural specialists. JOURNAL OF RURAL STUDIES. 2018;62:134–45.

204. Tse YK, Zhang M, Doherty B, Chappell P, Garnett P. Insight from the horsemeat scandal Exploring the consumers’ opinion of tweets toward Tesco. Industrial Management and Data Systems [Internet]. 2016;116(6):1178–200. Available from: https://www.scopus.com/inward/record.uri?eid=2-s2.0-84976584147&doi=10.1108%2fIMDS-10-2015-0417&partnerID=40&md5=5928f2b1ef2c9cd051c4bbcb98af0a9f

205. Tselengidis A, Östergren PO. Lobbying against sugar taxation in the European Union: Analysing the lobbying arguments and tactics of stakeholders in the food and drink industries. Scandinavian Journal of Public Health [Internet]. 2019;47(5):565–75. Available from: https://www.scopus.com/inward/record.uri?eid=2-s2.0-85049776324&doi=10.1177%2f1403494818787102&partnerID=40&md5=53a30347922f0b5d9b95d5f6856c5f7a

206. Uchendu FN, Abolarin TO. Corrupt practices negatively influenced food security and live expectancy in developing countries. The Pan African medical journal. 2015;20:110–110.

207. Uchendu FN. Hunger influenced life expectancy in war-torn Sub-Saharan African countries. Journal of health, population, and nutrition [Internet]. 2018;37(1):11–11. Available from: http://www.pubmedcentral.nih.gov/articlerender.fcgi?artid=PMC5921790

208. Ujunwa A, Okoyeuzu C, Kalu EU. Armed Conflict and Food Security in West Africa: Socioeconomic Perspective. INTERNATIONAL JOURNAL OF SOCIAL ECONOMICS. 2019;46(2):182–98.

209. Uzel G, Ndimballan A, Gurluk S. Impacts of Corruption to Agricultural Export Potential of the Gambia Competitive Neighbours. KSU TARIM VE DOGA DERGISI-KSU JOURNAL OF AGRICULTURE AND NATURE. 2021;24(4):886–94.

210. Vaidya R. Corruption, re-corruption and what transpires in between: The case of a government officer in India. Journal of Business Ethics. 2019;156(3):605–20.

211. Van Hoi P, Mol A, Oosterveer P. State governance of pesticide use and trade in Vietnam. NJAS - Wageningen Journal of Life Sciences [Internet]. 2013;67:19–26. Available from: https://www.scopus.com/inward/record.uri?eid=2-s2.0-84889880006&doi=10.1016%2fj.njas.2013.09.001&partnerID=40&md5=ada09a8094abb1105e1a98f013184ab1

212. Van Rijswijk W, Frewer LJ. Consumer needs and requirements for food and ingredient traceability information. International Journal of Consumer Studies. 2012;36(3):282–90.

213. van Ruth SM, van der Veeken J, Dekker P, Luning PA, Huisman W. Feeding fiction: Fraud vulnerability in the food service industry. Food Research International [Internet]. 2020;133. Available from: https://www.scopus.com/inward/record.uri?eid=2-s2.0-85081153496&doi=10.1016%2fj.foodres.2020.109158&partnerID=40&md5=3592ee3e9743474508db4db28f78bbce

214. van Ruth SM, Nillesen O. Which company characteristics make a food business at risk for food fraud? Foods [Internet]. 2021;10(4). Available from: https://www.scopus.com/inward/record.uri?eid=2-s2.0-85104120931&doi=10.3390%2ffoods10040842&partnerID=40&md5=c64c3318eae1b303c8a0510dbde62d48

215. van Velden JL, Travers H, Moyo BHZ, Biggs D. Using scenarios to understand community-based interventions for bushmeat hunting and consumption in African savannas. Biological Conservation [Internet]. 2020;248. Available from: https://www.scopus.com/inward/record.uri?eid=2-s2.0-85086937701&doi=10.1016%2fj.biocon.2020.108676&partnerID=40&md5=ffd204abbdb16794078a3b15a801fa8a

216. Vandenbrink D, Pauzé E, Potvin Kent M. Strategies used by the Canadian food and beverage industry to influence food and nutrition policies. The International Journal of Behavioral Nutrition and Physical Activity. 2020;17.

217. Venot JP, Andreini M, Pinkstaff CB. Planning and corrupting water resources development: The case of small reservoirs in Ghana. Water Alternatives [Internet]. 2011;4(3):399–423. Available from: https://www.scopus.com/inward/record.uri?eid=2-s2.0-83755196052&partnerID=40&md5=09317337f4ceb758a9b15106c041759e

218. Venter O, Sanderson EW, Magrach A, Allan JR, Beher J, Jones KR, et al. Sixteen years of change in the global terrestrial human footprint and implications for biodiversity conservation. Nature Communications [Internet]. 2016;7. Available from: https://www.scopus.com/inward/record.uri?eid=2-s2.0-84984638802&doi=10.1038%2fncomms12558&partnerID=40&md5=32607402866bd6705338806697ac528c

219. Vercillo S, Hird-Younger M. Farmer resistance to agriculture commercialisation in northern Ghana. THIRD WORLD QUARTERLY. 2019;40(4):763–79.

220. Verter N. Food Security and Trade in Food Products in Nigeria. European Journal of Sustainable Development. 2019;8(3):527–527.

221. Vollrath TL, Hallahan CB, Gehlhar MJ. Consumer demand and cost factors shape the global trade network in commodity and manufactured foods. Canadian Journal of Agricultural Economics. 2006;54(4):497–511.

222. Weber SA, Anders SM. Price rigidity and market power in German retailing. Managerial & Decision Economics. 2007;28(7):737–49.

223. Weesie R, García AK. From herding to farming under adaptation interventions in southern Kenya: A critical perspective. Sustainability (Switzerland) [Internet]. 2018;10(12). Available from: https://www.scopus.com/inward/record.uri?eid=2-s2.0-85057241251&doi=10.3390%2fsu10124386&partnerID=40&md5=629b5eda6379e313d555987ad0c49628

224. Wisniewski A, Buschulte A. How to tackle food fraud in official food control authorities in Germany. Journal fur Verbraucherschutz und Lebensmittelsicherheit [Internet]. 2019;14(4):319–28. Available from: https://www.scopus.com/inward/record.uri?eid=2-s2.0-85067290019&doi=10.1007%2fs00003-019-01228-2&partnerID=40&md5=a9a525efd015fe71ec5c0a4b6980e681

225. Wolfersberger J, Delacote P, Garcia S. An empirical analysis of forest transition and land-use change in developing countries. Ecological Economics [Internet]. 2015 Nov 1 [cited 2022 May 20];119:241–51. Available from: https://www.sciencedirect.com/science/article/pii/S0921800915003663

226. Yami M, van Asten P, Hauser M, Schut M, Pali P. Participation without Negotiating: Influence of Stakeholder Power Imbalances and Engagement Models on Agricultural Policy Development in Uganda. Rural Sociology [Internet]. 2019;84(2):390–415. Available from: https://www.scopus.com/inward/record.uri?eid=2-s2.0-85048919626&doi=10.1111%2fruso.12229&partnerID=40&md5=ba5bb36b214ab8d75073f9dc063c0489

227. Yan J, Erasmus SW, Aguilera Toro M, Huang H, van Ruth SM. Food fraud: Assessing fraud vulnerability in the extra virgin olive oil supply chain. Food Control [Internet]. 2020;111. Available from: https://www.scopus.com/inward/record.uri?eid=2-s2.0-85078725092&doi=10.1016%2fj.foodcont.2019.107081&partnerID=40&md5=ab863e6269b894cd107f23e3111cfbec

228. Yang Y, Huisman W, Hettinga KA, Zhang L, van Ruth SM. The Chinese milk supply chain: A fraud perspective. Food Control [Internet]. 2020;113. Available from: https://www.scopus.com/inward/record.uri?eid=2-s2.0-85081039321&doi=10.1016%2fj.foodcont.2020.107211&partnerID=40&md5=682cafeb09bdfb38c9e47084034efd77

229. Yankson PWK, Owusu AB, Frimpong S. Challenges and Strategies for Improving the Agricultural Marketing Environment in Developing Countries: Evidence From Ghana. Journal of Agricultural and Food Information [Internet]. 2016;17(1):49–61. Available from: https://www.scopus.com/inward/record.uri?eid=2-s2.0-84957800353&doi=10.1080%2f10496505.2015.1110030&partnerID=40&md5=1faa6bad620ce70029ab3aa51e782a86

230. Yasuda JK. Why Food Safety Fails in China: The Politics of Scale. CHINA QUARTERLY. 2015;223:745–69.

231. Yee WH, Liu P. Control, Coordination, and Capacity: Deficits in China?s Frontline Regulatory System for Food Safety. JOURNAL OF CONTEMPORARY CHINA.

232. Yengoh GT, Steen K, Armah FA, Ness B. Factors of vulnerability: How large-scale land acquisitions take advantage of local and national weaknesses in Sierra Leone. LAND USE POLICY. 2016;50:328–40.

233. Yu HH, Edmunds M, Lora-Wainwright A, Thomas D. Governance of the irrigation commons under integrated water resources management - A comparative study in contemporary rural China. Environmental Science and Policy [Internet]. 2016;55(P1):65–74. Available from: https://www.scopus.com/inward/record.uri?eid=2-s2.0-84941889631&doi=10.1016%2fj.envsci.2015.08.001&partnerID=40&md5=41a3e94a1d943c4eba23f5c52bd55b4d

234. Yunusa IA, Zerihun A, Gibberd MR. Analysis of the nexus between population, water resources and Global Food Security highlights significance of governance and research investments and policy priorities. Journal of the science of food and agriculture [Internet]. 2018;98(15):5764–75. Available from: http://www.ncbi.nlm.nih.gov/pubmed/29749117

235. Zhang W, Xue J. Economically motivated food fraud and adulteration in China: An analysis based on 1553 media reports. Food Control [Internet]. 2016;67:192–8. Available from: https://www.scopus.com/inward/record.uri?eid=2-s2.0-84959932120&doi=10.1016%2fj.foodcont.2016.03.004&partnerID=40&md5=fd0ad70c34a879c3685e29f579408657

236. Zhang Y, Pang M, Dickens BL, Edwards DP, Carrasco LR. Global hotspots of conversion risk from multiple crop expansion. Biological Conservation [Internet]. 2021;254. Available from: https://www.scopus.com/inward/record.uri?eid=2-s2.0-85099635768&doi=10.1016%2fj.biocon.2021.108963&partnerID=40&md5=71b27cc1390b6769f69726b242cf5467

237. Zhuang W, Liu M, Gao Z. A new method for quantifying the value of ecological environment damage caused by illegal fishing: A case study. Marine Pollution Bulletin [Internet]. 2021;172. Available from: https://www.scopus.com/inward/record.uri?eid=2-s2.0-85112474006&doi=10.1016%2fj.marpolbul.2021.112819&partnerID=40&md5=a746ce497124a20ca56814e9bb58018b

238. Kassem HS, Alotaibi BA. Do farmers perceive risks of fraudulent pesticides? Evidence from Saudi Arabia. PLoS ONE. 2020;15(9).

239. Perone G. The impact of agribusiness crimes on food prices: evidence from Italy. ECONOMIA POLITICA.

240. Rezazade F, Summers J, Lai Teik DO. A holistic approach to food fraud vulnerability assessment. Food Control [Internet]. 2022;131. Available from: https://www.scopus.com/inward/record.uri?eid=2-s2.0-85111113596&doi=10.1016%2fj.foodcont.2021.108440&partnerID=40&md5=48769d808f8e7180cca150ab19e0023f
